# Genetic diversity and population structure analysis in a large collection of white clover (*Trifolium repens* L.) germplasm worldwide

**DOI:** 10.7717/peerj.11325

**Published:** 2021-05-03

**Authors:** Feifei Wu, Sainan Ma, Jie Zhou, Chongyang Han, Ruchang Hu, Xinying Yang, Gang Nie, Xinquan Zhang

**Affiliations:** Department of Grassland Science, Animal Science and Technology College, Sichuan Agricultural University, Chengdu, Sichuan, China

**Keywords:** White clover, SSR, Genetic variation, Population structure

## Abstract

White clover is an important temperate legume forage with high nutrition. In the present study, 448 worldwide accessions were evaluated for the genetic variation and polymorphisms using 22 simple sequence repeat (SSR) markers. All the markers were highly informative, a total of 341 scored bands were amplified, out of which 337 (98.83%) were polymorphic. The PIC values ranged from 0.89 to 0.97 with an average of 0.95. For the AMOVA analysis, 98% of the variance was due to differences within the population and the remaining 2% was due to differences among populations. The white clover accessions were divided into different groups or subgroups based on PCoA, UPGMA, and STRUCTURE analyses. The existence of genetic differentiation between the originally natural and introduced areas according to the PCoA analysis of the global white clover accessions. There was a weak correlation between genetic relationships and geographic distribution according to UPGMA and STRUCTURE analyses. The results of the present study will provide the foundation for future breeding programs, genetic improvement, core germplasm collection establishment for white clover.

## Introduction

White clover (*Trifolium repens* L.) is a cool-season, allotetraploid (2n=4x=32) perennial legume species (Cogan et al., 2006; Isobe et al., 2012). It can grow well in a wide range of soil and environmental conditions with proper management, and it has extended its range globally by wild and cultivated distribution from its natural range (Europe, Western Asia, and North Africa) (Griffiths et al., 2019). It is an important companion species in perennial grass pastures in temperate latitudes for its high nutritional quality and strong nitrogen fixation ability (Barrett et al., 2004; Brink et al., 1999; George et al., 2006; Randazzo, Rosso & Pagano, 2013; Zhang et al., 2010). White clover is an obligate outcrossing species and shows strong gametophytic self-incompatibility, which leads to high genetic heterozygosity in populations (Aasmo Finne, Rognli & Schjelderup, 2000; Zhang et al., 2010).

Evaluation of genetic variation is essential for plant genetic resources conservation, selecting the genetically divergent parents for practice breeding and preventing genetic bases erosion of breeding populations (Dolanská & Čurn, 2004; Kölliker, Jones & Forster, 2001). Initial breeding efforts of white clover began in the 1930s and substantial genetic improvement has been achieved over the last 60–70 years (Zhang et al., 2010). As an outbreeding species, genetic improvement of white clover is always depending on mass or recurrent selection and based on polycross among multiple parental genotypes (George et al., 2006). White clover shows rich genetic diversity on many traits, such as leaf marks, cyanogenesis, herbage yield and leaf size. Although white clover is primarily propagated through clonal growth, high levels of genetic variation also could be detected in the populations of white clover (Gustine et al., 2002).

Genetic variation is frequently detected using morphological and agronomic characters, which often show multigenic inheritance with a strong influence by environmental factors. Molecular marker analysis offers an efficient alternative to this approach (Kölliker, Jones & Forster, 2001). Genetic variation of white clover has been studied using random amplified polymorphic DNA (RAPD) markers (Gustine et al., 2002; Zhang et al., 2010), amplified fragment length polymorphism (AFLP) (Kölliker, Jones & Forster, 2001; Van Treuren et al., 2005), restriction fragment length polymorphism (RFLP) (Dolanská & Čurn, 2004). Although the above markers could detect abundant genetic variation, however, the poor consistency, low reproducibility or elaborate operation limit their effectiveness (Roodt, Spies & Burger, 2002; Vos et al., 1995). In contrast, SSRs are codominant, high polymorphic, and multi-allelic genetic markers (Li et al., 2002). The markers associate with the non-repetitive regions of the genome and exhibit high mutation rates (Kalia et al., 2011; Morgante, Hanafey & Powell, 2002). SSR markers have been widely used to evaluate the genetic variation for various plants, e.g., *Medicago truncatula* (Eujayl et al., 2004), *Lolium multiflorum* (Nie et al., 2019), *Rhdodendron arboretum* (Sharma et al., 2020), *Pteroceltis tatarinowii* (Zhang et al., 2020).

SSR markers have also been applicated in white clover successfully, such as develop SSR markers for white clover (Kölliker et al., 2001) and used to evaluate genetic diversity (George et al., 2006; Randazzo, Rosso & Pagano, 2013; Zhang et al., 2010) and construct genetic linkage maps (Barrett et al., 2004; Griffiths et al., 2013; Isobe et al., 2012; Jones et al., 2003; Zhang et al., 2008; Zhang, Sledge & Bouton, 2007). The dendrogram employing SSR data of ten white clover germplasm collections from China showed the closest agreement with geographical origins (Zhang et al., 2010). Cultivars from New Zealand were more distant from the other cultivars based on SSR data (Randazzo, Rosso & Pagano, 2013). DNA fingerprints have been constructed for 10 commercial white clover cultivars by SSR markers (Ma et al., 2020), which showed that SSR markers are of great significance for the identification of special materials and could provide a basis for future studies of the genetic background. The genetic variation of white clover has also been evaluated by other technological means. The cluster analysis of 52 cultivars and accessions based on AFLP data only partially reflected their geographic origin (Kölliker, Jones & Forster, 2001). Eight white clover populations derived from different climates and geographic regions of North American showed high genetic similarities which indicated they have a common European origin (Gustine et al., 2002). As high informative molecular markers, SSRs can accelerate breeding programs greatly (Kölliker et al., 2001).

In the present study, 22 microsatellite markers (Griffiths et al., 2013; Griffiths et al., 2019) were used to evaluate the genetic variation among 448 white clover accessions collected from globally diverse origins. We analyzed the genetic diversity among accessions in terms of geographical origin. Our results have important implications for future breeding, germplasm improvement, and core germplasm collection in white clover.

## Materials and Methods

### Plant materials

**Figure 1:**
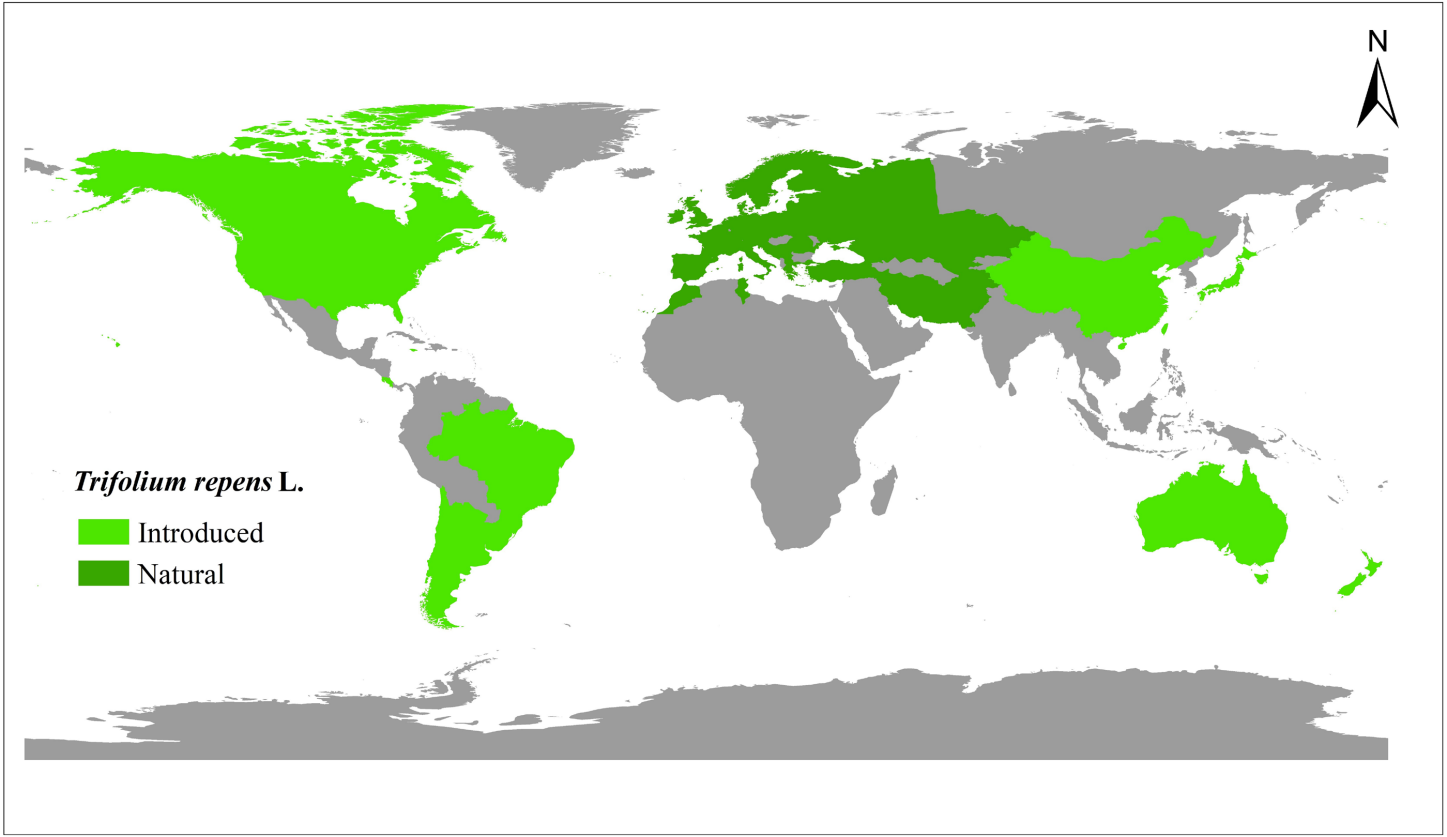
The distribution of 448 white clover (*Trifolium repens* L.) accessions included in the present study.

A total of 448 white clover accessions were collected from the worldwide range (Fig. 1, Table S1) (Daday, 1958; Griffiths et al., 2019). The seeds were obtained from the Margot Forde Forage Germplasm Centre (New Zealand), National Plant Germplasm System (United States of America), National Herbage Germplasm Conservation Centre of China, Institute of Grasslands Research of CAAS (Chinese Academy of Agricultural Sciences), and Institute of Animal Sciences of CAAS. All the materials are currently maintained at Chongzhou (103.644°E, 30.560°N), Sichuan, China.

### DNA extraction and SSRs-PCR

The total DNA was extracted from fresh leaf samples using a DNA Extraction kit (Tiangen Biotech Co., Beijing, China). SSRs primers developed in previous studies (Griffiths et al., 2013) were used in the present study. In all, 22 primers (supplied by Sangon Biotech Co., Shanghai, China) were used in the analysis (Table S2). SSRs-PCR amplification reactions were carried out in 20 µL volumes, containing 1 µL genomic DNA (50 ng), 12.5 µL 2 × Taq PCR mix (Tiangen Biotech Co., Beijing, China), 2 µL primers (1 µL forward primer and 1 µL reverse primer) and ddH_2_O to adjust the volume. The PCR program was carried out as follows: 94 °C for 5 min, followed by 35 cycles of 94 °C for 1 min, 55 °C for 30s, and 72 °C for 40s, and a final extension at 72 °C for 10 min. The PCR products were examined using 8.0% polyacrylamide gels electrophoresis under 400 volts for 2 h and were visualized using silver staining.

### Data scoring and statistical analysis

The amplification bands were scored for the presence (1) or absence (0) and a binary matrix was formed for SSR markers. The total number of bands (TNB), number of polymorphic bands (NPB) and percentage of polymorphic bands (PPB) were calculated. Polymorphic information content (PIC) was calculated using the formula PIC = 1 − ∑ *P*_*i*_^2^, and the *P*_*i*_ is the frequency of the *i-*th allele (Powell et al., 1996). The number of polymorphic loci (NPL), the percentage of polymorphic loci (PPL), the observed number of alleles (*Na*), the effective number of alleles (*Ne*), Nei’s (1973) gene diversity (*h*), and Shannon’s information index (*I*) were calculated by GenAlEx 6.5 (Peakall & Smouse, 2012) to evaluate the genetic diversity within accessions and populations.

Genetic distance, the principal coordinate analysis (PCoA) and the analysis of molecular variance (AMOVA) were conducted using GenAlEx 6.5 (Peakall & Smouse, 2012). The unweighted pair-group method with arithmetic means (UPGMA) cluster analysis was performed based on Nei’s unbiased genetic distance matrix with MEGA X (Kumar et al., 2018). Population genetic structure was determined using the model-based program in the STRUCTURE 2.3.4 software with a Bayesian approach (Falush, Stephens & Pritchard, 2003; Falush, Stephens & Pritchard, 2007). The number of the most likelihood populations (K) was tested for 1–10 and 10 interactions were done for each K. The 500,000 initial burn-in replications were followed by 100,000 Markov Chain Monte Carlo (MCMC) replications. The optimal K capturing the major structure in the white clover data was determined using Structure Harvester (http://taylor0.biology.ucla.edu/structureHarvester/) (Earl & vonHoldt, 2012; Evanno, Regnaut & Goudet, 2005).

## Results

### The polymorphism of SSR markers

In this study, a total of 341 scored bands were amplified using 22 SSR primers across 448 accessions, out of which 337 (98.83%) were polymorphic (Table 1). The number of polymorphic bands for each primer combination varied from 7 (gtrs1113) to 25 (gtrs749), with an average of 15.30 bands. All the primers had a high PIC value and identified a high level of polymorphism. The percentage of polymorphic bands revealed different levels of polymorphisms ranging from 91.67% to 100%. And the PIC values ranged from 0.89 to 0.97 with an average of 0.95. The primers also showed high Nei’s genetic diversity (*h*) and Shannon’s Information index (*I*). The *h* is ranged from 0.198 to 0.345 with an average of 0.280, and the *I* is ranged from 0.339 to 0.520 with an average of 0.437 (Table 1).

**Table 1:** Polymorphism analysis of 448 white clover accessions with SSR primers.

Primers	TNB	NPB	PPB (%)	PIC	*h*	*I*
ats002	21	21	100	0.97	0.272	0.425
gtrs1113	7	7	100	0.89	0.198	0.339
gtrs165	13	13	100	0.95	0.207	0.352
gtrs171	12	12	100	0.95	0.345	0.520
gtrs173	15	15	100	0.95	0.246	0.388
gtrs211	12	12	100	0.94	0.287	0.442
gtrs242	14	14	100	0.96	0.336	0.509
gtrs285	22	22	100	0.97	0.270	0.419
gtrs292	12	12	100	0.95	0.278	0.436
gtrs319	19	19	100	0.97	0.301	0.472
gtrs371	10	10	100	0.93	0.302	0.460
gtrs376	17	17	100	0.96	0.262	0.415
gtrs541	13	13	100	0.95	0.340	0.504
gtrs564	12	11	91.67	0.94	0.251	0.398
gtrs591	11	11	100	0.94	0.277	0.435
gtrs679	20	20	100	0.97	0.295	0.464
gtrs701	23	22	95.65	0.97	0.236	0.388
gtrs723	16	15	93.75	0.96	0.300	0.455
gtrs749	25	25	100	0.97	0.309	0.477
gtrs760	12	11	91.67	0.95	0.264	0.417
gtrs851	18	18	100	0.97	0.345	0.515
gtrs949	17	17	100	0.96	0.229	0.375
Mean	15.50	15.32	98.76	0.95	0.280	0.437

**Notes.**

Note TNB Total number of bands NPB Number of polymorphic bands PPB Percentage of polymorphic bands PIC polymorphism information content *h* Nei’s (1973) gene diversity *I* Shannon’s Information index [Lewontin (1972)]

### Genetic diversity analysis

The genetic diversity was analyzed for the natural and introduced groups (Table 2). The percentage of polymorphic loci of the natural group (98.24%) is higher than the introduced (96.77%). The number of polymorphic loci values is 335 and 330 respectively. The observed number of alleles of the natural group (1.982) is also higher than the introduced (1.956), as well as the effective number of alleles, which is 1.450 and 1.433 respectively. The Nei’s gene diversity values for the natural group are 0.280 and 0.273 for the introduced group. Correspondingly, the higher Shannon’s information index was recorded for the natural group (0.437) and the lower for the introduced group (0.427).

**Table 2:** Genetic variability within 448 white clover accessions detected by SSR markers.

Populations		Accessions number	NPL	PPL (%)	Na	Ne	*h*	*I*
Natural	European	144	334	97.95	1.977 ± 0.009	1.449 ± 0.017	0.279 ± 0.008	0.434 ± 0.010
	Asian and Russia	54	316	92.67	1.891 ± 0.022	1.442 ± 0.018	0.269 ± 0.009	0.415 ± 0.012
	Mediterranean	57	326	95.60	1.935 ± 0.017	1.419 ± 0.016	0.266 ± 0.008	0.418 ± 0.010
	Mean			95.41	1.935 ± 0.010	1.437 ± 0.010	0.271 ± 0.005	0.422 ± 0.006
Introduced	America	42	325	95.31	1.933 ± 0.017	1.434 ± 0.017	0.268 ± 0.009	0.416 ± 0.011
	Australia	102	335	98.24	1.979 ± 0.009	1.442 ± 0.016	0.278 ± 0.008	0.434 ± 0.010
	Asian	49	324	95.01	1.930 ± 0.018	1.423 ± 0.017	0.265 ± 0.008	0.413 ± 0.011
	Mean			96.19	1.947 ± 0.009	1.433 ± 0.010	0.270 ± 0.005	0.421 ± 0.006
All	natural	255	335	98.24	1.982 ± 0.007	1.450 ± 0.016	0.280 ± 0.008	0.437 ± 0.010
	Introduced	193	330	96.77	1.956 ± 0.014	1.433 ± 0.016	0.273 ± 0.008	0.427 ± 0.010
	Mean			97.51	1.969 ± 0.008	1.441 ± 0.011	0.277 ± 0.006	0.432 ± 0.007

**Notes.**

Note NPL the number of polymorphic loci PPL the percentage of polymorphic loci Na observed number of alleles Ne effective number of alleles *h* Nei’s (1973) gene diversity *I* Shannon’s Information index [Lewontin (1972)]

The genetic diversity index also is calculated in the subgroups (Table 3). The NPL values for subgroups ranged from 316 (Asian from Natural) to 335 (Australia from Introduced). The highest PPL was 98.24% was recorded in Australia from the introduced group, while the lowest was 92.67% for Asian from the natural group. The *Na* ranged from 1.891 in subgroup Asian from the natural group and 1.979 from subgroup Australia from the introduced group. The Ne varies from 1.419 to 1.449, which was recorded in the Mediterranean and Europe from the natural group. The Nei’s gene diversity values for subgroups ranged from 0.265 (Asian from Introduced) to 0.279 (European from Natural). Correspondingly, the highest *I* was recorded for subgroup European (0.434) and Australia (0.434), and the lowest for subgroup Asian from Introduced (0.413).

**Table 3:** Analysis of molecular variance (AMOVA) for 448 accessions of white clover.

Source of variance		Degrees of freedom	Sum of squares	Mean square	Variance components	Total variance (%)	*P*-value
Natural range	Among populations	2	405.509	202.755	1.938	3%	<0.05
	Within populations	252	14,578.977	57.853	57.853	97%	<0.05
	Total	254	14,984.486		59.791	100%	<0.05
Introduced range	Among populations	2	360.087	180.043	2.072	3%	<0.05
	Within populations	190	11,072.680	58.277	58.277	97%	<0.05
	Total	192	11,432.767		60.350	100%	<0.05
All accessions	Among populations	1	283.142	283.142	1.022	2%	<0.05
	Within populations	446	26,293.630	58.954	58.954	98%	<0.05
	Total	447	26,576.772		59.976	100%	<0.05

Analysis of molecular variance (AMOVA) was implemented to evaluate variance components among groups and subgroups (Table 3), which showed highly significant differences (*P* < 0.05). Of the total accessions, 98% of the variance was due to differences among the accessions within the groups and the remaining 2% was due to differences between the groups. Of the natural group, 97% of the variance was due to differences among the accessions within the subgroups and the remaining 3% was due to differences among the subgroups. It showed the same result in the AMOVA analysis of the introduced group, 97% differences showed among the accessions and 3% showed among the subgroups.

### Cluster and population structure analysis

**Figure 2:**
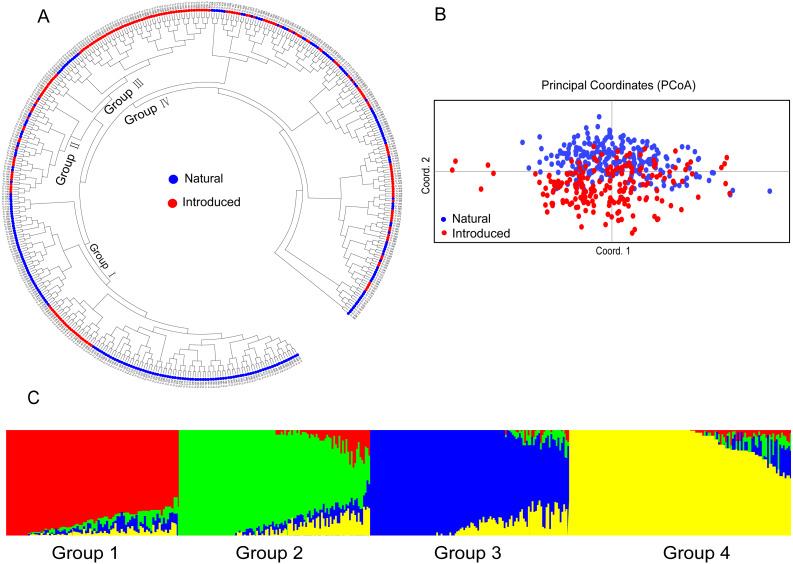
The UPGMA (A) PCoA (B) and STRUCTURE analysis (C) among 448 white clover accessions.

The relationship among the accessions from the different groups and subgroups based on genetic distance was further determined by UPGMA cluster analysis, PCoA analysis, and genetic structure analysis. Clear population differentiation is absent in UPGMA using scored SSR markers in this study, and each group contained accessions of various sources in population structure analysis. According to the UPGMA dendrogram (Fig. 2A), all the accessions from the natural and introduced range could be classified into four clusters (Fig. 2A). The accessions from the natural group and the introduced group could be divided into different subclades in cluster I and cluster III. In cluster I, 24 accessions from the introduced range clustered into one subclade and all belong to subgroup Australia. While the accessions from the natural range come from the subgroup European. In the cluster III, 12 accessions from the natural range clustered into one subclade and come from subgroup European. Meanwhile, the accessions from the introduced range mainly come from Asia and Australia. In cluster II and cluster IV, the accessions from the natural and introduced range were closely related. Further, the subclades span the extremes of the dendrogram were the accessions from the natural range. The UPGMA dendrogram of all the accessions showed that the Australia and Asia accessions (introduced) had a closer genetic relationship with the European accessions (natural) (Cluster I & III, Fig. 2A). And the American accessions (introduced) may be closed to the Mediterranean accessions (Cluster II & IV, Fig. 2A). The genetic distance (Table S3) between two Asia accessions (Tr_058 and Tr_059) was the least, while the largest genetic distance was showed between Europe (Tr_252) and Australia (Tr_318) accessions. According to the PCoA analysis, all the accessions could be classified as natural and introduced populations (Fig. 2B). The PCoA of SSR data grouped the accessions as the natural and introduced range (Fig. 2B). Structure software was run for K = 2–10 based on the distribution of the SSR data among the 448 accessions. Based on maximum likelihood and delta K (Δ*K*) values, the number of optimum groups was four (Fig. 2C and Fig. S1). Among them, Group 1 contained 98 accessions, of which 51 accessions come from the introduced range, it showed that the 51 accessions had a close relationship with the other 47 natural accessions. Group 2 contained 109 accessions (93 natural accessions and 16 introduced accessions), Which almost all the natural accessions. Group 3 contained 113 accessions (43 introduced accessions and 70 natural accessions), and Group 4 contained 128 accessions (82 introduced accessions and 46 natural accessions). The genetic structure revealed most accessions with admixture in each group, while accessions in group 4 showed less admixture.

**Figure 3:**
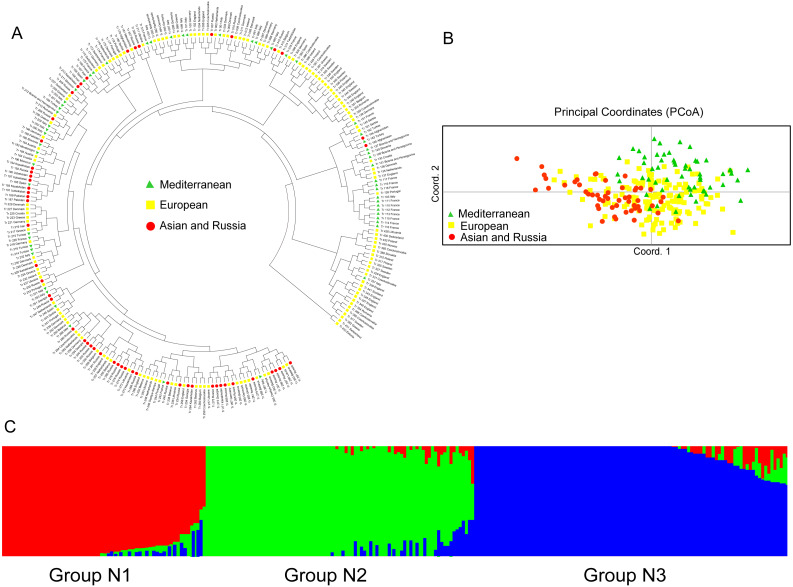
The UPGMA (A) PCoA (B) and STRUCTURE analysis (C) among 255 natural white clover accessions.

The UPGMA dendrogram of the natural accessions showed that the accessions from Europe were distributed throughout the dendrogram (Fig. 3A). The accessions of Asia and Russia (European) mainly clustered at one end, and most of the Mediterranean mainly clustered at the other end. The Mediterranean accessions had further genetic distance with the accessions from Asia (Fig. 3A). The PCoA analysis showed a clustering pattern synonymous with the UPGMA dendrogram (Fig. 3B). Structure software was run for K = 2–10 based on the distribution of the SSR data among the 255 accessions. Based on maximum likelihood and delta K (Δ*K*) values, the number of optimum groups was three (Fig. 3C and Fig. S2). Among them, Group N1 contained 66 accessions (43 accessions from subgroup European; 17 accessions from subgroup Asia and Russia; 6 accessions from subgroup Mediterranean). Group N2 contained 87 accessions (32 accessions from subgroup European; 31 accessions from subgroup Asia and Russia; 24 accessions from subgroup Mediterranean). The remained 102 accessions were assigned to Group N3 (69 accessions from subgroup European; 6 accessions from subgroup Asia and Russia; 27 accessions from subgroup Mediterranean). The genetic structure revealed most accessions with admixture in each group while accessions in group 3 showed less admixture, which mostly comes from Europe.

**Figure 4:**
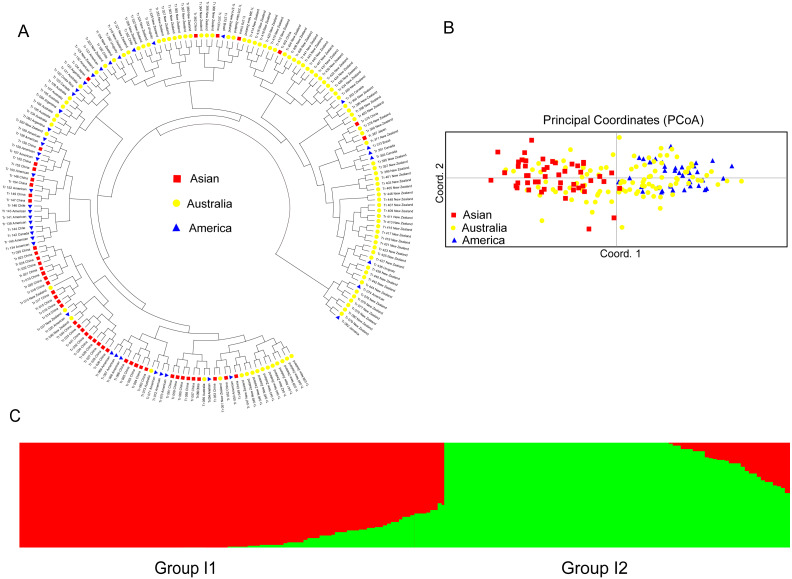
The UPGMA (A) PCoA (B) and STRUCTURE analysis (C) among 193 introduced white clover accessions.

For the accessions from the introduced range, the subgroup Asian accessions mainly clustered in one clade, which also clustered with several American and Australian accessions. Most of the American accessions also clustered within one clade. The Australia accessions were distributed all through the dendrogram (Fig. 4A). The PCoA analysis of the introduced accessions showed that the Asian accessions could separate from the American accessions. All the above two subgroup accessions were mixed with the Australia accessions (Fig. 4B). Structure software was run for K = 2–10 based on the distribution of the SSR data among the 193 accessions. Based on maximum likelihood and delta K (Δ*K*) values, the number of optimum groups was two (Fig. 4C and Fig. S3). Group I1 contained 106 accessions (31 accessions from subgroup America; 32 accessions from subgroup Australia; 43 accessions from subgroup Asian). The remained 87 accessions were assigned to Group I2 (11 accessions from subgroup America; 70 accessions from subgroup Australia; 6 accessions from subgroup Asian). The genetic structure of the introduced accessions revealed less admixture than the natural accessions.

## Discussion

### Marker polymorphism and genetic diversity analysis

Evaluation of genetic diversity for outbreeding forage species is important for breeding improvement (Dolanská & Čurn, 2004). White clover is a highly heterogeneous and outbreeding species (Cogan et al., 2006; Isobe et al., 2012), substantial genetic variation among the white clover accessions was observed as expected. In the present study, all the 22 SSR markers showed highly polymorphic. The mean PIC value (0.95) was higher than the values of the primers used in the study of Kölliker et al. (2001) and George et al. (2006), which were 0.68 and 0.66. It is even higher than other genus and species, based on SSRs data, such as genus *Melilotus* with 0.87 (Wu et al., 2016) and alfalfa with 0.608 (Wang et al., 2013). This may be on account of the SSR markers are more polymorphic as codominant markers (Griffiths et al., 2013; Wu et al., 2016). It also might result from the different environments (geographical origin) of the 448 accessions and a high percentage of outcrossing in the species.

White clover has a high level of genetic heterogeneity within natural and synthetic populations (George et al., 2006; Williams, Baker & Williams, 1987). In this study, the genetic diversity of the natural population (*h* = 0.280, *I* = 0.437) was slightly more evident than that of the introduced population (*h* = 0.273, *I* = 0.427). The high-level genetic diversity partly because the two diploid progenitors of white clover come from very different environments (extreme coastal or alpine habitats) (Griffiths et al., 2019), and partly because of multiple introduction events of white clover. Among natural subpopulations, the European had the highest level of genetic diversity (*h* = 0.279, *I* = 0.434) which was due to the European region was the origin of white clover. The Australian subpopulation had a higher level of genetic diversity (*h* = 0.278, *I* = 0.434) than the other two introduced subpopulations. This suggested the Australia accessions may have more diverse sources, and multiple introductions from different regions resulted in high genetic diversity in Australia.

Genetic variation between the populations (97%–98%) was higher than that within populations (2%–3%) in the present study. The result is consistent with the previous studies of white clover based on RAPD (73% within population) (Gustine & Huff, 1999), AFLP analysis (84% within cultivars) (Kölliker, Jones & Forster, 2001) and SSR (86.5% within cultivars) (George et al., 2006). Which also consistent with the other outcrossing species, such as perennial ryegrass (Bolaric et al., 2005; Van Treuren et al., 2005). The high intrapopulation variability was attributed to the allogamous reproductive behavior, and the variation of white clover mainly comes from the intrapopulation variation.

### Population genetic structure of white clover germplasm resources

White clover is a successful allotetraploid example of allopolyploidy-facilitated niche expansion, which has facilitated global radiation of the previously confined specialist progenitor genomes (Griffiths et al., 2019). It is considered that the indigenous area consists of the whole European, North Africa (Morocco and Tunisia) and the western half of the Asiatic distribution area. Moreover, the species has invaded globally through the animal, human and spontaneous distribution (Daday, 1958). In our study, the existence of genetic differentiation between the originally natural and introduced areas according to the PCoA analysis of the global white clover accessions. It is similar to the results of Jahufer et al. (2003), the clustering of white clover cultivars also indicated a strong correlation with geographic origin based on EST-SSRs analysis. Cluster analysis of 52 white clover accessions based on the AFLP data also showed a partial association between cultivar groups and geographic origin (Kölliker, Jones & Forster, 2001).

In contrast, clear population differentiation with the geographic origin was absent in UPGMA and STRUCTURE analyses, in which no group exclusively included the accessions from a single region. The results were consistent with George et al. (2006), who found no obvious distinction among white clover accessions among the geographical origins. The weak correlation between genetic relationships and geographic distribution conforms with the reports in *Eruca sativa* (Golkar & Bakhtiari, 2020), *Vicia faba* (Ammar et al., 2015) and *Camellia sinensis* (Zhang et al., 2018). It may be attributed that there is no significant correlation between genetic distance and geographical distance (Golkar & Nourbakhsh, 2019). In the present study, the UPGMA dendrogram of all the accessions showed that the clusters have substantial overlap of different populations. Moreover, high values of the genetic mixture were also confirmed by STRUCTURE analysis. It is largely due to the outcrossing and self-incompatibility of plant species (Khan et al., 2009), human seed transplantation (Daday, 1958; Wang et al., 2009), different biological dispersal patterns and evolutionary forces (Chapman et al., 2010) and random dispersal in a region (Golkar & Mokhtari, 2018). The given genetic admixture of white clover may result from a complicated hybrid ancestry, and the high rate of outcrossing could result in genetic admixture from adjacent regions (Griffiths et al., 2019).

White clover spread by natural means to the largest part of the Asiatic mainland (Daday, 1958). It was supported by our results, which showed that the least genetic distance existed in Asia accessions. The level of genetic diversity was also the lowest among all the subpopulations. Moreover, white clover was carried to introduced Japan (Asia) from Dutch (Europe) in 1846. The Japanese accession also gathered with European accessions to a subclade in the present study. According to the references (Daday, 1958; Gustine et al., 2002), white clover was introduced into America and Australia from Europe. However, the largest genetic distance was showed between Europe and Australia accessions. It suggested that the introduced white clover adapted to new environments by forming genetic variation. The genetic diversity of the European subpopulations from the natural range were at a pretty high level in our results. The abundant genetic variation could provide an excellent genetic basis for practice breeding. Hence, the European collections, especially the coastal and the alpine area (Griffiths et al., 2019), could be recommended as alternative collections for core germplasm collections selection. The core collections should maintain the vast majority of germplasm diversity (Lv et al., 2020), and the optimal fraction of core collection for white clover needs to be further studied.

The white clover accessions in the present study were divided into different groups or subgroups based on PCoA, UPGMA and STRUCTURE analyses. It could be attributed to the different statistical principles (Gower, 1966; Lv et al., 2020; Pritchard et al., 2000). PCoA can provide a more valid classification based on the dissimilarity matrix of the original data, which is not strict with the Hardy-Weinberg equilibrium assumption. STRUCTURE assigns the accessions to subgroup probabilistically by a Bayesian clustering approach, and it is always used for the subdivision of natural out-crossing populations. And the accessions were clustered using UPGMA analysis is implemented based on genetic distance, which showed more detailed relationships among the accessions. Overall, these three methods could work together to provide a comprehensive understanding of the white clover population genetic structure.

In conclusion, the findings of the study confirmed that global white clover accessions contained a high level of genetic diversity. And the weak correlation between genetic relationships and geographic distribution of white clover accessions. Our result will provide molecular evidence for breeding improvement, germplasm resources conservation and core germplasm collection establishment for white clover.

##  Supplemental Information

10.7717/peerj.11325/supp-1 Table S1 The detail information of the materials in the present study

10.7717/peerj.11325/supp-2 Table S2 The detail information of primers included in the study

10.7717/peerj.11325/supp-3 Table S3 The genetic distance of the 448 white clover accessions

10.7717/peerj.11325/supp-4 Figure S1 The STRUCTURE analysis among 448 white clover accessions

10.7717/peerj.11325/supp-5 Figure S2 The STRUCTURE analysis among 255 natural white clover accessions

10.7717/peerj.11325/supp-6 Figure S3 The STRUCTURE analysis among 193 introduced white clover accessions

## Additional Information and Declarations

### Data Availability

The following information was supplied regarding data availability: The raw measurements, material availability and accessions are available in the Supplemental Files.

## References

[ref-1] Ammar MH, Alghamdi SS, Migdadi HM, Khan MA, El-Harty EH, Al-Faifi SA (2015). Assessment of genetic diversity among faba bean genotypes using agro-morphological and molecular markers. Saudi Journal of Biological Sciences.

[ref-2] Aasmo Finne M, Rognli OA, Schjelderup I (2000). Genetic variation in a Norwegian germplasm collection of white clover (Trifolium repens L.). Euphytica.

[ref-3] Barrett B, Griffiths A, Schreiber M, Ellison N, Mercer C, Bouton J, Ong B, Forster J, Sawbridge T, Spangenberg G, Bryan G, Woodfield D (2004). A microsatellite map of white clover. Theoretical and Applied Genetics.

[ref-4] Bolaric S, Barth S, Melchinger AE, Posselt UK (2005). Genetic diversity in European perennial ryegrass cultivars investigated with RAPD markers. Plant Breeding.

[ref-5] Brink GE, Pederson GA, Alison MW, Ball DM, Bouton JH (1999). Growth of white clover ecotypes, cultivars, and germplasms in the southeastern USA. Crop Science.

[ref-6] Chapman MA, Hvala J, Strever J, Burke JM (2010). Population genetic analysis of safflower (Carthamus tinctorius; Asteraceae) reveals a Near Eastern origin and five centers of diversity. American Journal of Botany.

[ref-7] Cogan NO, Abberton MT, Smith KF, Kearney G, Marshall AH, Williams A, Michaelson-Yeates TP, Bowen C, Jones ES, Vecchies AC, Forster JW (2006). Individual and multi-environment combined analyses identify QTLs for morphogenetic and reproductive development traits in white clover (*Trifolium repens* L.). Theoretical and Applied Genetics.

[ref-8] Daday H (1958). Gene frequencies in wild populations of Trifolium repens L. III. World Distribution. Heredity.

[ref-9] Dolanská L, Čurn V (2004). Identification of white clover (Trifolium repens L.) cultivars using molecular markers. Plant Soil and Environment.

[ref-10] Earl DA, Von Holdt BM (2012). STRUCTURE HARVESTER: a website and program for visualizing STRUCTURE output and implementing the Evanno method. Conservation Genetics Resources.

[ref-11] Eujayl I, Sledge MK, Wang L, May GD, Chekhovskiy K, Zwonitzer JC, Mian MA (2004). *Medicago truncatula* EST-SSRs reveal cross-species genetic markers for Medicago spp. Theoretical and Applied Genetics.

[ref-12] Evanno G, Regnaut S, Goudet J (2005). Detecting the number of clusters of individuals using the software STRUCTURE: a simulation study. Molecular Ecology.

[ref-13] Falush D, Stephens M, Pritchard JK (2003). Inference of population structure using multilocus genotype data: linked loci and correlated allele frequencies. Genetics.

[ref-14] Falush D, Stephens M, Pritchard JK (2007). Inference of population structure using multilocus genotype data: dominant markers and null alleles. Molecular Ecology Notes.

[ref-15] George J, Dobrowolski MP, Van Zijllde Jong E, Cogan NO, Smith KF, Forster JW (2006). Assessment of genetic diversity in cultivars of white clover (Trifolium repens L.) detected by SSR polymorphisms. Genome.

[ref-16] Golkar P, Bakhtiari MA (2020). Evaluation of genetic diversity in the world collection of *Eruca sativa* L. using oil content, fatty acids and molecular markers. Industrial Crops and Products.

[ref-17] Golkar P, Mokhtari N (2018). Molecular diversity assessment of a world collection of safflower genotypes by SRAP and SCoT molecular markers. Physiology and Molecular Biology of Plants.

[ref-18] Golkar P, Nourbakhsh V (2019). Analysis of genetic diversity and population structure in Nigella sativa L. using agronomic traits and molecular markers (SRAP and SCoT). Industrial Crops and Products.

[ref-19] Gower JC (1966). Some distance properties of latent root and vector methods used in multivariate analysis. Biometrika.

[ref-20] Griffiths AG, Barrett BA, Simon D, Khan AK, Bickerstaff P, Anderson CB, Franzmayr BK, Hancock KR, Jones CS (2013). An integrated genetic linkage map for white clover (Trifolium repens L.) with alignment to Medicago. BMC Genomics.

[ref-21] Griffiths AG, Moraga R, Tausen M, Gupta V, Bilton TP, Campbell MA, Ashby RL, Nagy I, Khan A, Larking A, Anderson C, Franzmayr B, Hancock K, Scott A, Ellison NW, Cox M, Asp T, Mailund T, Schierup MH, Andersen SU (2019). Breaking free: the genomics of allopolyploidy-facilitated niche expansion in white clover. The Plant Cell.

[ref-22] Gustine DL, Huff DR (1999). Genetic Variation within among White Clover Populations from Managed Permanent Patures of the Northeastern USA. Crop ence.

[ref-23] Gustine DL, Voigt PW, Brummer EC, Papadopoulos YA (2002). Genetic variation of RAPD markers for North American white clover collections and cultivars. Crop Science.

[ref-24] Isobe SN, Hisano H, Sato S, Hirakawa H, Okumura K, Shirasawa K, Sasamoto S, Watanabe A, Wada T, Kishida Y, Tsuruoka H, Fujishiro T, Yamada M, Kohara M, Tabata S (2012). Comparative genetic mapping and discovery of linkage disequilibrium across linkage groups in white clover (Trifolium repens L.). G3-Genes Genomes Genetics.

[ref-25] Jahufer Z, Barrett B, Griffiths A, Woodfield D (2003). DNA fingerprinting and genetic relationships among white clover cultivars. Proceedings of the New Zealand Grassland Association.

[ref-26] Jones ES, Hughes LJ, Drayton MC, Abberton MT, Michaelsonyeates TPT, Bowen C, Forster JW (2003). An SSR and AFLP molecular marker-based genetic map of white clover (Trifolium repens L.). Plant Science.

[ref-27] Kalia RK, Rai MK, Kalia S, Singh R, Dhawan AK (2011). Microsatellite markers: an overview of the recent progress in plants. Euphytica.

[ref-28] Khan MA, Von Witzke-Ehbrecht S, Maass BL, Becker HC (2009). Relationships among different geographical groups, agro-morphology, fatty acid composition and RAPD marker diversity in Safflower (Carthamus tinctorius). Genetic Resources and Crop Evolution.

[ref-29] Kölliker R, Jones ES, Drayton MC, Dupal MP, Forster JW (2001). Development and characterisation of simple sequence repeat (SSR) markers for white clover (Trifolium repens L.). Theoretical and Applied Genetics.

[ref-30] Kölliker R, Jones ES, Forster MZZJW (2001). Bulked AFLP analysis for the assessment of genetic diversity in white clover (Trifolium repens L.). Euphytica.

[ref-31] Kumar S, Stecher G, Li M, Knyaz C, Tamura K (2018). MEGA X: molecular evolutionary genetics analysis across computing platforms. Molecular Biology and Evolution.

[ref-32] Li YC, Korol AB, Fahima T, Beiles A, Nevo E (2002). Microsatellites: genomic distribution, putative functions and mutational mechanisms: a review. Molecular Ecology.

[ref-33] Lv J, Li C, Zhou C, Chen J, Li F, Weng Q, Li M, Wang Y, Chen S, Chen J, Gan S (2020). Genetic diversity analysis of a breeding population of Eucalyptus cloeziana F. Muell. (Myrtaceae) and extraction of a core germplasm collection using microsatellite markers. Industrial Crops and Products.

[ref-34] Ma S, Han C, Zhou J, Hu R, Jiang X, Wu F, Tian K, Nie G, Zhang X (2020). Fingerprint identification of white clover cultivars based on SSR molecular markers. Molecular Biology Reports.

[ref-35] Morgante M, Hanafey M, Powell W (2002). Microsatellites are preferentially associated with nonrepetitive DNA in plant genomes. Nature Genetics.

[ref-36] Nie G, Huang T, Ma X, Huang L, Peng Y, Yan Y, Li Z, Wang X, Zhang X (2019). Genetic variability evaluation and cultivar identification of tetraploid annual ryegrass using SSR markers. PeerJ.

[ref-37] Peakall R, Smouse PE (2012). GenAlEx 6.5: genetic analysis in Excel. Population genetic software for teaching and research—an update. Bioinformatics.

[ref-38] Powell W, Morgante M, Andre C, Hanafey M, Vogel J, Tingey S, Rafalski A (1996). The comparison of RFLP, RAPD, AFLP and SSR (microsatellite) markers for germplasm analysis. Molecular Breeding.

[ref-39] Pritchard JK, Stephens M, Rosenberg NA, Donnelly P (2000). Association mapping in structured populations. American Journal of Human Genetics.

[ref-40] Randazzo CP, Rosso BS, Pagano EM (2013). Identification of white clover cultivars (*Trifolium repens* L.) using SSR. Bag Journal of Basic & Applied Genetics.

[ref-41] Roodt R, Spies J, Burger T (2002). Preliminary DNA fingerprinting of the turf grass *Cynodon dactylon* (Poaceae: Chloridoideae). Bothalia.

[ref-42] Sharma H, Kumar P, Singh A, Aggarwal K, Roy J, Sharma V, Rawat S (2020). Development of polymorphic EST-SSR markers and their applicability in genetic diversity evaluation in Rhododendron arboreum. Molecular Biology Reports.

[ref-43] Van Treuren R, Bas N, Goossens PJ, Jansen J, Van Soest LJ (2005). Genetic diversity in perennial ryegrass and white clover among old Dutch grasslands as compared to cultivars and nature reserves. Molecular Ecology.

[ref-44] Vos P, Hogers R, Bleeker M, Reijans M, Lee TVD, Hornes M, Friters A, Pot J, Paleman J, Kuiper M, Zabeau M (1995). AFLP: a new technique for DNA fingerprinting. Nucleic Acids Research.

[ref-45] Wang Z, Yan H, Fu X, Li X, Gao H (2013). Development of simple sequence repeat markers and diversity analysis in alfalfa (Medicago sativa L.). Molecular Biology Reports.

[ref-46] Wang Z, Yuan X, Zheng Y, Liu J (2009). Molecular identification and genetic analysis for 24 turf-type Cynodon cultivars by Sequence-Related Amplified Polymorphism markers. Scientia Horticulturae.

[ref-47] Williams WM, Baker MJ, Williams WM (1987). White clover taxonomy and biosystematics. White Clover.

[ref-48] Wu F, Zhang D, Ma J, Luo K, Di H, Liu Z, Zhang J, Wang Y (2016). Analysis of genetic diversity and population structure in accessions of the genus Melilotus. Industrial Crops and Products.

[ref-49] Zhang Y, He J, Zhao PX, Bouton JH, Monteros MJ (2008). Genome-wide identification of microsatellites in white clover (Trifolium repens L.) using FIASCO and phpSSRMiner. Plant Methods.

[ref-50] Zhang Y, Sledge MK, Bouton JH (2007). Genome mapping of white clover (Trifolium repens L.) and comparative analysis within the Trifolieae using cross-species SSR markers. Theoretical and Applied Genetics.

[ref-51] Zhang Y, Zhang X, Chen X, Sun W, Li J (2018). Genetic diversity and structure of tea plant in Qinba area in China by three types of molecular markers. Hereditas.

[ref-52] Zhang M, Zhang Y, Wang G, Zhou J, Tian Y, Geng Q, Wang Z (2020). Development and characterization of 20 novel EST-SSR markers for *Pteroceltis tatarinowii*, a relict tree in China. Applications in Plant Sciences.

[ref-53] Zhang X, Zhang YJ, Yan R, Han JG, Hong F, Wang JH, Cao K (2010). Genetic variation of white clover (Trifolium repens L.) collections from China detected by morphological traits, RAPD and SSR. African Journal of Biotechnology.

