# Supplementary material for: Genetic diversity and population structure analysis in a large collection of white clover (Trifolium repens L.) germplasm worldwide
Source: PeerJ. 2021 May 3;9:e11325. doi: 10.7717/peerj.11325 (PMC8101478; doi:10.7717/peerj.11325)
Supplement: Table S1 [file peerj-09-11325-s001.docx]

| **Code** | **Materials ID** | **Origin** | **Seed sources** |
| --- | --- | --- | --- |
| TR-001 | CF 022386 | Italy | National Herbage Germplasm Conservation Centre of China |
| TR-002 | CF 001320 | Netherlands | National Herbage Germplasm Conservation Centre of China |
| TR-003 | CF 022360 | Denmark | National Herbage Germplasm Conservation Centre of China |
| TR-004 | CF 022346 | Netherlands | National Herbage Germplasm Conservation Centre of China |
| TR-005 | CF 022376 | Czech | National Herbage Germplasm Conservation Centre of China |
| TR-006 | CF 022383 | England | National Herbage Germplasm Conservation Centre of China |
| TR-007 | CF 022392 | Russia | National Herbage Germplasm Conservation Centre of China |
| TR-008 | CF 022345 | Denmark | National Herbage Germplasm Conservation Centre of China |
| TR-009 | CF 022375 | Czech | National Herbage Germplasm Conservation Centre of China |
| TR-010 | CF 030373 | Russia | National Herbage Germplasm Conservation Centre of China |
| TR-011 | CF 000806 | Denmark | National Herbage Germplasm Conservation Centre of China |
| TR-012 | CF 022401 | Ireland | National Herbage Germplasm Conservation Centre of China |
| TR-013 | CF 000051 | New Zealand | National Herbage Germplasm Conservation Centre of China |
| TR-014 | CF 025851 | China | National Herbage Germplasm Conservation Centre of China |
| TR-015 | CF 022601 | China | National Herbage Germplasm Conservation Centre of China |
| TR-016 | CF 022600 | China | National Herbage Germplasm Conservation Centre of China |
| TR-017 | CF 022558 | China | National Herbage Germplasm Conservation Centre of China |
| TR-018 | CF 022547 | China | National Herbage Germplasm Conservation Centre of China |
| TR-019 | CF 022446 | China | National Herbage Germplasm Conservation Centre of China |
| TR-020 | CF 022445 | China | National Herbage Germplasm Conservation Centre of China |
| TR-021 | CF 022444 | China | National Herbage Germplasm Conservation Centre of China |
| TR-022 | CF 022440 | China | National Herbage Germplasm Conservation Centre of China |
| TR-023 | CF 022422 | China | National Herbage Germplasm Conservation Centre of China |
| TR-024 | CF 022421 | China | National Herbage Germplasm Conservation Centre of China |
| TR-025 | CF 000804 | China | National Herbage Germplasm Conservation Centre of China |
| TR-026 | CF 022415 | China | National Herbage Germplasm Conservation Centre of China |
| TR-027 | CF 022368 | China | National Herbage Germplasm Conservation Centre of China |
| TR-028 | CF 022367 | China | National Herbage Germplasm Conservation Centre of China |
| TR-029 | CF 022352 | China | National Herbage Germplasm Conservation Centre of China |
| TR-030 | CF 022351 | China | National Herbage Germplasm Conservation Centre of China |
| TR-031 | CF 022341 | China | National Herbage Germplasm Conservation Centre of China |
| TR-032 | CF 006900 | China | National Herbage Germplasm Conservation Centre of China |
| TR-033 | CF 006887 | China | National Herbage Germplasm Conservation Centre of China |
| TR-034 | CF 006886 | China | National Herbage Germplasm Conservation Centre of China |
| TR-035 | CF 005852 | America | National Herbage Germplasm Conservation Centre of China |
| TR-036 | CF 005851 | New Zealand | National Herbage Germplasm Conservation Centre of China |
| TR-037 | CF 005848 | New Zealand | National Herbage Germplasm Conservation Centre of China |
| TR-038 | CF 005844 | New Zealand | National Herbage Germplasm Conservation Centre of China |
| TR-039 | CF 005843 | New Zealand | National Herbage Germplasm Conservation Centre of China |
| TR-040 | CF 005841 | New Zealand | National Herbage Germplasm Conservation Centre of China |
| TR-041 | CF 005840 | New Zealand | National Herbage Germplasm Conservation Centre of China |
| TR-042 | CF 005839 | New Zealand | National Herbage Germplasm Conservation Centre of China |
| TR-043 | CF 005838 | New Zealand | National Herbage Germplasm Conservation Centre of China |
| TR-044 | CF 005837 | New Zealand | National Herbage Germplasm Conservation Centre of China |
| TR-045 | CF 005836 | New Zealand | National Herbage Germplasm Conservation Centre of China |
| TR-046 | CF 005835 | New Zealand | National Herbage Germplasm Conservation Centre of China |
| TR-047 | CF 005834 | New Zealand | National Herbage Germplasm Conservation Centre of China |
| TR-048 | CF 005832 | New Zealand | National Herbage Germplasm Conservation Centre of China |
| TR-049 | CF 005812 | China | National Herbage Germplasm Conservation Centre of China |
| TR-050 | CF 002737 | America | National Herbage Germplasm Conservation Centre of China |
| TR-051 | CF 000868 | New Zealand | National Herbage Germplasm Conservation Centre of China |
| TR-052 | CF 000864 | China | National Herbage Germplasm Conservation Centre of China |
| TR-053 | CF 000810 | China | National Herbage Germplasm Conservation Centre of China |
| TR-054 | CF 000108 | America | National Herbage Germplasm Conservation Centre of China |
| TR-055 | CF 000056 | China | National Herbage Germplasm Conservation Centre of China |
| TR-056 | CF 000053 | Australia | National Herbage Germplasm Conservation Centre of China |
| TR-057 | 04667 | China | Institute of Grasslands Research of CAAS (Chinese Academy of Agricultural Sciences) |
| TR-058 | 02613 | China | Institute of Grasslands Research of CAAS (Chinese Academy of Agricultural Sciences) |
| TR-059 | 02483 | China | Institute of Grasslands Research of CAAS (Chinese Academy of Agricultural Sciences) |
| TR-060 | 01775 | China | Institute of Grasslands Research of CAAS (Chinese Academy of Agricultural Sciences) |
| TR-061 | 01457 | China | Institute of Grasslands Research of CAAS (Chinese Academy of Agricultural Sciences) |
| TR-062 | 01453 | China | Institute of Grasslands Research of CAAS (Chinese Academy of Agricultural Sciences) |
| TR-063 | 01232 | China | Institute of Grasslands Research of CAAS (Chinese Academy of Agricultural Sciences) |
| TR-064 | 01176 | China | Institute of Grasslands Research of CAAS (Chinese Academy of Agricultural Sciences) |
| TR-065 | 01044 | China | Institute of Grasslands Research of CAAS (Chinese Academy of Agricultural Sciences) |
| TR-066 | 00605 | China | Institute of Grasslands Research of CAAS (Chinese Academy of Agricultural Sciences) |
| TR-067 | NSL 186524 | America | National Plant Germplasm System (United States of America) |
| TR-068 | NSL 30343 | America | National Plant Germplasm System (United States of America) |
| TR-069 | NSL 30344 | America | National Plant Germplasm System (United States of America) |
| TR-070 | NSL 30354 | America | National Plant Germplasm System (United States of America) |
| TR-071 | NSL 32652 | America | National Plant Germplasm System (United States of America) |
| TR-072 | NSL 5459 | America | National Plant Germplasm System (United States of America) |
| TR-073 | NSL 5462 | Australia | National Plant Germplasm System (United States of America) |
| TR-074 | NSL 7505 | America | National Plant Germplasm System (United States of America) |
| TR-075 | PI 100247 | New Zealand | National Plant Germplasm System (United States of America) |
| TR-076 | PI 100250 | New Zealand | National Plant Germplasm System (United States of America) |
| TR-077 | PI 100251 | New Zealand | National Plant Germplasm System (United States of America) |
| TR-078 | PI 161363 | New Zealand | National Plant Germplasm System (United States of America) |
| TR-079 | PI 190125 | New Zealand | National Plant Germplasm System (United States of America) |
| TR-080 | PI 195530 | New Zealand | National Plant Germplasm System (United States of America) |
| TR-081 | PI 195535 | Italy | National Plant Germplasm System (United States of America) |
| TR-082 | PI 197830 | Jamaica | National Plant Germplasm System (United States of America) |
| TR-083 | PI 197870 | Argentina | National Plant Germplasm System (United States of America) |
| TR-084 | PI 202069 | Argentina | National Plant Germplasm System (United States of America) |
| TR-085 | PI 215809 | Denmark | National Plant Germplasm System (United States of America) |
| TR-086 | PI 217444 | Italy | National Plant Germplasm System (United States of America) |
| TR-087 | PI 217510 | Denmark | National Plant Germplasm System (United States of America) |
| TR-088 | PI 232110 | Germany | National Plant Germplasm System (United States of America) |
| TR-089 | PI 232112 | Germany | National Plant Germplasm System (United States of America) |
| TR-090 | PI 233746 | Italy | National Plant Germplasm System (United States of America) |
| TR-091 | PI 233813 | Italy | National Plant Germplasm System (United States of America) |
| TR-092 | PI 237199 | Netherlands | National Plant Germplasm System (United States of America) |
| TR-093 | PI 237200 | Netherlands | National Plant Germplasm System (United States of America) |
| TR-094 | PI 237291 | Denmark | National Plant Germplasm System (United States of America) |
| TR-095 | PI 237733 | Germany | National Plant Germplasm System (United States of America) |
| TR-096 | PI 237734 | Germany | National Plant Germplasm System (United States of America) |
| TR-097 | PI 241460 | Italy | National Plant Germplasm System (United States of America) |
| TR-098 | PI 260646 | Greece | National Plant Germplasm System (United States of America) |
| TR-099 | PI 282375 | Italy | National Plant Germplasm System (United States of America) |
| TR-100 | PI 282376 | Germany | National Plant Germplasm System (United States of America) |
| TR-101 | PI 282377 | Italy | National Plant Germplasm System (United States of America) |
| TR-102 | PI 291833 | England | National Plant Germplasm System (United States of America) |
| TR-103 | PI 291837 | Italy | National Plant Germplasm System (United States of America) |
| TR-104 | PI 291842 | Australia | National Plant Germplasm System (United States of America) |
| TR-105 | PI 291843 | Australia | National Plant Germplasm System (United States of America) |
| TR-106 | PI 291844 | Australia | National Plant Germplasm System (United States of America) |
| TR-107 | PI 291845 | Australia | National Plant Germplasm System (United States of America) |
| TR-108 | PI 291846 | Australia | National Plant Germplasm System (United States of America) |
| TR-109 | PI 291847 | Australia | National Plant Germplasm System (United States of America) |
| TR-110 | PI 294542 | France | National Plant Germplasm System (United States of America) |
| TR-111 | PI 294543 | France | National Plant Germplasm System (United States of America) |
| TR-112 | PI 294544 | France | National Plant Germplasm System (United States of America) |
| TR-113 | PI 294545 | France | National Plant Germplasm System (United States of America) |
| TR-114 | PI 294546 | France | National Plant Germplasm System (United States of America) |
| TR-115 | PI 294547 | France | National Plant Germplasm System (United States of America) |
| TR-116 | PI 294548 | France | National Plant Germplasm System (United States of America) |
| TR-117 | PI 294551 | France | National Plant Germplasm System (United States of America) |
| TR-118 | PI 294555 | France | National Plant Germplasm System (United States of America) |
| TR-119 | PI 294558 | France | National Plant Germplasm System (United States of America) |
| TR-120 | PI 296380 | Canada | National Plant Germplasm System (United States of America) |
| TR-121 | PI 303837 | Australia | National Plant Germplasm System (United States of America) |
| TR-122 | PI 304154 | Costa Rica | National Plant Germplasm System (United States of America) |
| TR-123 | PI 306286 | Argentina | National Plant Germplasm System (United States of America) |
| TR-124 | PI 420000 | Japan | National Plant Germplasm System (United States of America) |
| TR-125 | PI 517508 | New Zealand | National Plant Germplasm System (United States of America) |
| TR-126 | PI 517509 | Portugal | National Plant Germplasm System (United States of America) |
| TR-127 | PI 542849 | Bosnia and Herzegovina | National Plant Germplasm System (United States of America) |
| TR-128 | PI 542901 | Bosnia and Herzegovina | National Plant Germplasm System (United States of America) |
| TR-129 | PI 542902 | Slovenia | National Plant Germplasm System (United States of America) |
| TR-130 | PI 542906 | Croatia | National Plant Germplasm System (United States of America) |
| TR-131 | PI 542907 | Bosnia and Herzegovina | National Plant Germplasm System (United States of America) |
| TR-132 | PI 564537 | America | National Plant Germplasm System (United States of America) |
| TR-133 | PI 583854 | America | National Plant Germplasm System (United States of America) |
| TR-134 | PI 583855 | America | National Plant Germplasm System (United States of America) |
| TR-135 | PI 595910 | Netherlands | National Plant Germplasm System (United States of America) |
| TR-136 | PI 595915 | Denmark | National Plant Germplasm System (United States of America) |
| TR-137 | PI 595920 | England | National Plant Germplasm System (United States of America) |
| TR-138 | PI 595921 | Germany | National Plant Germplasm System (United States of America) |
| TR-139 | PI 595925 | America | National Plant Germplasm System (United States of America) |
| TR-140 | PI 596568 | America | National Plant Germplasm System (United States of America) |
| TR-141 | PI 597492 | America | National Plant Germplasm System (United States of America) |
| TR-142 | PI 597494 | Canada | National Plant Germplasm System (United States of America) |
| TR-143 | PI 597504 | America | National Plant Germplasm System (United States of America) |
| TR-144 | PI 597509 | Chile | National Plant Germplasm System (United States of America) |
| TR-145 | PI 597517 | Serbia | National Plant Germplasm System (United States of America) |
| TR-146 | PI 597528 | Chile | National Plant Germplasm System (United States of America) |
| TR-147 | PI 611656 | China | National Plant Germplasm System (United States of America) |
| TR-148 | PI 611660 | China | National Plant Germplasm System (United States of America) |
| TR-149 | PI 611661 | China | National Plant Germplasm System (United States of America) |
| TR-150 | PI 631875 | America | National Plant Germplasm System (United States of America) |
| TR-151 | PI 631883 | France | National Plant Germplasm System (United States of America) |
| TR-152 | PI 631892 | America | National Plant Germplasm System (United States of America) |
| TR-153 | PI 631902 | China | National Plant Germplasm System (United States of America) |
| TR-154 | PI 634071 | China | National Plant Germplasm System (United States of America) |
| TR-155 | PI 634192 | China | National Plant Germplasm System (United States of America) |
| TR-156 | PI 641529 | China | National Plant Germplasm System (United States of America) |
| TR-157 | PI 655609 | America | National Plant Germplasm System (United States of America) |
| TR-158 | PI 655618 | America | National Plant Germplasm System (United States of America) |
| TR-159 | PI 655619 | America | National Plant Germplasm System (United States of America) |
| TR-160 | PI 655620 | America | National Plant Germplasm System (United States of America) |
| TR-161 | PI 655884 | Serbia | National Plant Germplasm System (United States of America) |
| TR-162 | PI 204510 | Turkey | National Plant Germplasm System (United States of America) |
| TR-163 | PI 205062 | Turkey | National Plant Germplasm System (United States of America) |
| TR-164 | PI 220837 | Afghanistan | National Plant Germplasm System (United States of America) |
| TR-165 | PI 221962 | Afghanistan | National Plant Germplasm System (United States of America) |
| TR-166 | PI 223021 | Iran | National Plant Germplasm System (United States of America) |
| TR-167 | PI 227255 | Iran | National Plant Germplasm System (United States of America) |
| TR-168 | PI 234489 | Spain | National Plant Germplasm System (United States of America) |
| TR-169 | PI 234677 | France | National Plant Germplasm System (United States of America) |
| TR-170 | PI 234680 | France | National Plant Germplasm System (United States of America) |
| TR-171 | PI 234840 | Germany | National Plant Germplasm System (United States of America) |
| TR-172 | PI 234936 | Switzerland | National Plant Germplasm System (United States of America) |
| TR-173 | PI 234938 | Switzerland | National Plant Germplasm System (United States of America) |
| TR-174 | PI 234961 | France | National Plant Germplasm System (United States of America) |
| TR-175 | PI 235116 | Denmark | National Plant Germplasm System (United States of America) |
| TR-176 | PI 235126 | Netherlands | National Plant Germplasm System (United States of America) |
| TR-177 | PI 239978 | Portugal | National Plant Germplasm System (United States of America) |
| TR-178 | PI 239979 | Portugal | National Plant Germplasm System (United States of America) |
| TR-179 | PI 250790 | Afghanistan | National Plant Germplasm System (United States of America) |
| TR-180 | PI 251190 | Serbia | National Plant Germplasm System (United States of America) |
| TR-181 | PI 251191 | Macedonia | National Plant Germplasm System (United States of America) |
| TR-182 | PI 251194 | Montenegro | National Plant Germplasm System (United States of America) |
| TR-183 | PI 251432 | Croatia | National Plant Germplasm System (United States of America) |
| TR-184 | PI 251862 | Austria | National Plant Germplasm System (United States of America) |
| TR-185 | PI 251863 | Austria | National Plant Germplasm System (United States of America) |
| TR-186 | PI 253323 | Slovenia | National Plant Germplasm System (United States of America) |
| TR-187 | PI 269980 | Pakistan | National Plant Germplasm System (United States of America) |
| TR-188 | PI 269983 | Pakistan | National Plant Germplasm System (United States of America) |
| TR-189 | PI 269990 | Pakistan | National Plant Germplasm System (United States of America) |
| TR-190 | PI 314342 | Uzbekistan | National Plant Germplasm System (United States of America) |
| TR-191 | PI 314345 | Uzbekistan | National Plant Germplasm System (United States of America) |
| TR-192 | PI 314588 | Russia | National Plant Germplasm System (United States of America) |
| TR-193 | PI 314762 | Kazakhstan | National Plant Germplasm System (United States of America) |
| TR-194 | PI 314763 | Kazakhstan | National Plant Germplasm System (United States of America) |
| TR-195 | PI 319139 | Spain | National Plant Germplasm System (United States of America) |
| TR-196 | PI 384699 | Morocco | National Plant Germplasm System (United States of America) |
| TR-197 | PI 388632 | Uzbekistan | National Plant Germplasm System (United States of America) |
| TR-198 | PI 414366 | Germany | National Plant Germplasm System (United States of America) |
| TR-199 | PI 418750 | Turkey | National Plant Germplasm System (United States of America) |
| TR-200 | PI 418910 | Italy | National Plant Germplasm System (United States of America) |
| TR-201 | PI 418911 | Italy | National Plant Germplasm System (United States of America) |
| TR-202 | PI 418912 | Italy | National Plant Germplasm System (United States of America) |
| TR-203 | PI 418917 | Italy | National Plant Germplasm System (United States of America) |
| TR-204 | PI 418918 | Italy | National Plant Germplasm System (United States of America) |
| TR-205 | PI 419300 | Greece | National Plant Germplasm System (United States of America) |
| TR-206 | PI 419302 | Greece | National Plant Germplasm System (United States of America) |
| TR-207 | PI 419459 | Switzerland | National Plant Germplasm System (United States of America) |
| TR-208 | PI 440745 | Russia | National Plant Germplasm System (United States of America) |
| TR-209 | PI 440746 | Russia | National Plant Germplasm System (United States of America) |
| TR-210 | PI 494747 | Romania | National Plant Germplasm System (United States of America) |
| TR-211 | PI 517126 | Morocco | National Plant Germplasm System (United States of America) |
| TR-212 | PI 542915 | Bosnia and Herzegovina | National Plant Germplasm System (United States of America) |
| TR-213 | PI 634157 | Kazakhstan | National Plant Germplasm System (United States of America) |
| TR-214 | PI 655773 | Tunisia | National Plant Germplasm System (United States of America) |
| TR-215 | PI 655774 | Tunisia | National Plant Germplasm System (United States of America) |
| TR-216 | PI 655779 | Tunisia | National Plant Germplasm System (United States of America) |
| TR-217 | PI 249873 | Greece | National Plant Germplasm System (United States of America) |
| TR-218 | PI 367898 | Germany | National Plant Germplasm System (United States of America) |
| TR-219 | PI 227876 | Iran | National Plant Germplasm System (United States of America) |
| TR-220 | PI 234678 | France | National Plant Germplasm System (United States of America) |
| TR-221 | PI 234840 | Germany | National Plant Germplasm System (United States of America) |
| TR-222 | PI 288084 | Ireland | National Plant Germplasm System (United States of America) |
| TR-223 | PI 419304 | Greece | National Plant Germplasm System (United States of America) |
| TR-224 | PI 419316 | Greece | National Plant Germplasm System (United States of America) |
| TR-225 | PI 542904 | Croatia | National Plant Germplasm System (United States of America) |
| TR-226 | PI 634148 | Kazakhstan | National Plant Germplasm System (United States of America) |
| TR-227 | Kelangde | Denmark | National Plant Germplasm System (United States of America) |
| TR-228 | xiweite | Denmark | National Plant Germplasm System (United States of America) |
| TR-229 | Miligro | Denmark | National Plant Germplasm System (United States of America) |
| TR-230 | Haifa | Denmark | National Plant Germplasm System (United States of America) |
| TR-231 | PI 282378 | Italy | National Plant Germplasm System (United States of America) |
| TR-232 | PI 208567 | Italy | National Plant Germplasm System (United States of America) |
| TR-233 | zxy2010p-7404 | Portugal | Institute of Animal Sciences of CAAS (Chinese Academy of Agricultural Sciences) |
| TR-234 | zxy2010p-7143 | Russia | Institute of Animal Sciences of CAAS (Chinese Academy of Agricultural Sciences) |
| TR-235 | zxy2010p-7096 | Russia | Institute of Animal Sciences of CAAS (Chinese Academy of Agricultural Sciences) |
| TR-236 | zxy2010p-7808 | Lithuania | Institute of Animal Sciences of CAAS (Chinese Academy of Agricultural Sciences) |
| TR-237 | zxy2010p-7523 | Ukraine | Institute of Animal Sciences of CAAS (Chinese Academy of Agricultural Sciences) |
| TR-238 | zxy2010p-7513 | Belarus | Institute of Animal Sciences of CAAS (Chinese Academy of Agricultural Sciences) |
| TR-239 | zxy2010p-7308 | Germany | Institute of Animal Sciences of CAAS (Chinese Academy of Agricultural Sciences) |
| TR-240 | zxy2010p-7043 | Russia | Institute of Animal Sciences of CAAS (Chinese Academy of Agricultural Sciences) |
| TR-241 | zxy2010p-7398 | Portugal | Institute of Animal Sciences of CAAS (Chinese Academy of Agricultural Sciences) |
| TR-242 | zxy2010p-7339 | France | Institute of Animal Sciences of CAAS (Chinese Academy of Agricultural Sciences) |
| TR-243 | zxy2010p-7572 | Serbia | Institute of Animal Sciences of CAAS (Chinese Academy of Agricultural Sciences) |
| TR-244 | zxy2010p-7363 | Portugal | Institute of Animal Sciences of CAAS (Chinese Academy of Agricultural Sciences) |
| TR-245 | zxy2010p-7768 | Spain | Institute of Animal Sciences of CAAS (Chinese Academy of Agricultural Sciences) |
| TR-246 | zxy2010p-7860 | North Ossetia – Alania | Institute of Animal Sciences of CAAS (Chinese Academy of Agricultural Sciences) |
| TR-247 | zxy2010p-7559 | Netherlands | Institute of Animal Sciences of CAAS (Chinese Academy of Agricultural Sciences) |
| TR-248 | zxy2006p-2621 | Netherlands | Institute of Animal Sciences of CAAS (Chinese Academy of Agricultural Sciences) |
| TR-249 | zxy2006p-2405 | Russia | Institute of Animal Sciences of CAAS (Chinese Academy of Agricultural Sciences) |
| TR-250 | zxy2006p-1972 | Ukraine | Institute of Animal Sciences of CAAS (Chinese Academy of Agricultural Sciences) |
| TR-251 | zxy2007p-3109 | Georgia | Institute of Animal Sciences of CAAS (Chinese Academy of Agricultural Sciences) |
| TR-252 | zxy2007p-3322 | Belarus | Institute of Animal Sciences of CAAS (Chinese Academy of Agricultural Sciences) |
| TR-253 | zxy2007p-3769 | Italy | Institute of Animal Sciences of CAAS (Chinese Academy of Agricultural Sciences) |
| TR-254 | zxy2007p-3185 | Georgia | Institute of Animal Sciences of CAAS (Chinese Academy of Agricultural Sciences) |
| TR-255 | zxy2007p-4105 | Belarus | Institute of Animal Sciences of CAAS (Chinese Academy of Agricultural Sciences) |
| TR-256 | zxy2007p-3248 | England | Institute of Animal Sciences of CAAS (Chinese Academy of Agricultural Sciences) |
| TR-257 | zxy2007p-3692 | Netherlands | Institute of Animal Sciences of CAAS (Chinese Academy of Agricultural Sciences) |
| TR-258 | zxy2007p-3375 | Czech | Institute of Animal Sciences of CAAS (Chinese Academy of Agricultural Sciences) |
| TR-259 | zxy2007p-4043 | England | Institute of Animal Sciences of CAAS (Chinese Academy of Agricultural Sciences) |
| TR-260 | zxy2007p-4101 | Belgium | Institute of Animal Sciences of CAAS (Chinese Academy of Agricultural Sciences) |
| TR-261 | zxy2007p-4004 | Russia | Institute of Animal Sciences of CAAS (Chinese Academy of Agricultural Sciences) |
| TR-262 | zxy2007p-4105 | Belarus | Institute of Animal Sciences of CAAS (Chinese Academy of Agricultural Sciences) |
| TR-263 | zxy2007p-4130 | Belarus | Institute of Animal Sciences of CAAS (Chinese Academy of Agricultural Sciences) |
| TR-264 | zxy2007p-3777 | Kazakhstan | Institute of Animal Sciences of CAAS (Chinese Academy of Agricultural Sciences) |
| TR-265 | zxy2007p-3240 | Russia | Institute of Animal Sciences of CAAS (Chinese Academy of Agricultural Sciences) |
| TR-266 | zxy2007p-3300 | England | Institute of Animal Sciences of CAAS (Chinese Academy of Agricultural Sciences) |
| TR-267 | zxy2007p-3405 | Germany | Institute of Animal Sciences of CAAS (Chinese Academy of Agricultural Sciences) |
| TR-268 | zxy2007p-3020 | Russia | Institute of Animal Sciences of CAAS (Chinese Academy of Agricultural Sciences) |
| TR-269 | zxy2007p-3499 | Russia | Institute of Animal Sciences of CAAS (Chinese Academy of Agricultural Sciences) |
| TR-270 | zxy2007p-3842 | Kazakhstan | Institute of Animal Sciences of CAAS (Chinese Academy of Agricultural Sciences) |
| TR-271 | zxy2007p-3791 | Kazakhstan | Institute of Animal Sciences of CAAS (Chinese Academy of Agricultural Sciences) |
| TR-272 | zxy2007p-3556 | Lithuania | Institute of Animal Sciences of CAAS (Chinese Academy of Agricultural Sciences) |
| TR-273 | zxy2007p-3102 | Georgia | Institute of Animal Sciences of CAAS (Chinese Academy of Agricultural Sciences) |
| TR-274 | zxy2007p-4155 | Russia | Institute of Animal Sciences of CAAS (Chinese Academy of Agricultural Sciences) |
| TR-275 | zxy2007p-3230 | Russia | Institute of Animal Sciences of CAAS (Chinese Academy of Agricultural Sciences) |
| TR-276 | zxy2007p-3506 | Russia | Institute of Animal Sciences of CAAS (Chinese Academy of Agricultural Sciences) |
| TR-277 | zxy2007p-3550 | Ukraine | Institute of Animal Sciences of CAAS (Chinese Academy of Agricultural Sciences) |
| TR-278 | zxy2006p-2286 | Russia | Institute of Animal Sciences of CAAS (Chinese Academy of Agricultural Sciences) |
| TR-279 | zxy2006p-1768 | Russia | Institute of Animal Sciences of CAAS (Chinese Academy of Agricultural Sciences) |
| TR-280 | zxy2006p-1686 | Russia | Institute of Animal Sciences of CAAS (Chinese Academy of Agricultural Sciences) |
| TR-281 | zxy2006p-2304 | Sweden | Institute of Animal Sciences of CAAS (Chinese Academy of Agricultural Sciences) |
| TR-282 | zxy2006p-1513 | Netherlands | Institute of Animal Sciences of CAAS (Chinese Academy of Agricultural Sciences) |
| TR-283 | zxy2005p-863 | Ukraine | Institute of Animal Sciences of CAAS (Chinese Academy of Agricultural Sciences) |
| TR-284 | zxy2005p-1401 | Czech | Institute of Animal Sciences of CAAS (Chinese Academy of Agricultural Sciences) |
| TR-285 | zxy2006p-2128 | Denmark | Institute of Animal Sciences of CAAS (Chinese Academy of Agricultural Sciences) |
| TR-286 | zxy2006p-1711 | Russia | Institute of Animal Sciences of CAAS (Chinese Academy of Agricultural Sciences) |
| TR-287 | zxy2006p-1489 | Italy | Institute of Animal Sciences of CAAS (Chinese Academy of Agricultural Sciences) |
| TR-288 | zxy2006p-2344 | Italy | Institute of Animal Sciences of CAAS (Chinese Academy of Agricultural Sciences) |
| TR-289 | zxy2006p-1827 | Belarus | Institute of Animal Sciences of CAAS (Chinese Academy of Agricultural Sciences) |
| TR-290 | zxy2006p-1819 | Belarus | Institute of Animal Sciences of CAAS (Chinese Academy of Agricultural Sciences) |
| TR-291 | zxy2006p-1636 | Russia | Institute of Animal Sciences of CAAS (Chinese Academy of Agricultural Sciences) |
| TR-292 | zxy2006p-2180 | Denmark | Institute of Animal Sciences of CAAS (Chinese Academy of Agricultural Sciences) |
| TR-293 | zxy2006p-1879 | Belarus | Institute of Animal Sciences of CAAS (Chinese Academy of Agricultural Sciences) |
| TR-294 | zxy2006p-2093 | Georgia | Institute of Animal Sciences of CAAS (Chinese Academy of Agricultural Sciences) |
| TR-295 | zxy2006p-2528 | Latvia | Institute of Animal Sciences of CAAS (Chinese Academy of Agricultural Sciences) |
| TR-296 | zxy2005p-1388 | Germany | Institute of Animal Sciences of CAAS (Chinese Academy of Agricultural Sciences) |
| TR-297 | zxy2005p-879 | Russia | Institute of Animal Sciences of CAAS (Chinese Academy of Agricultural Sciences) |
| TR-298 | zxy2006p-1924 | Belgium | Institute of Animal Sciences of CAAS (Chinese Academy of Agricultural Sciences) |
| TR-299 | zxy2006p-605 | Russia | Institute of Animal Sciences of CAAS (Chinese Academy of Agricultural Sciences) |
| TR-300 | zxy2006p-2488 | Russia | Institute of Animal Sciences of CAAS (Chinese Academy of Agricultural Sciences) |
| TR-301 | zxy2006p-2475 | Russia | Institute of Animal Sciences of CAAS (Chinese Academy of Agricultural Sciences) |
| TR-302 | zxy2006p-1735 | Russia | Institute of Animal Sciences of CAAS (Chinese Academy of Agricultural Sciences) |
| TR-303 | zxy2006p-1798 | Belarus | Institute of Animal Sciences of CAAS (Chinese Academy of Agricultural Sciences) |
| TR-304 | zxy2006p-2496 | Russia | Institute of Animal Sciences of CAAS (Chinese Academy of Agricultural Sciences) |
| TR-305 | zxy2006p-2387 | North Ossetia – Alania | Institute of Animal Sciences of CAAS (Chinese Academy of Agricultural Sciences) |
| TR-306 | zxy2006p-1316 | England | Institute of Animal Sciences of CAAS (Chinese Academy of Agricultural Sciences) |
| TR-307 | zxy2006p-1754 | Russia | Institute of Animal Sciences of CAAS (Chinese Academy of Agricultural Sciences) |
| TR-308 | zxy2005p-1373 | Germany | Institute of Animal Sciences of CAAS (Chinese Academy of Agricultural Sciences) |
| TR-309 | zxy2006p-2508 | Russia | Institute of Animal Sciences of CAAS (Chinese Academy of Agricultural Sciences) |
| TR-310 | zxy2006p-2233 | Russia | Institute of Animal Sciences of CAAS (Chinese Academy of Agricultural Sciences) |
| TR-311 | zxy2006p-2007 | Ukraine | Institute of Animal Sciences of CAAS (Chinese Academy of Agricultural Sciences) |
| TR-312 | zxy2006p-1918 | Belgium | Institute of Animal Sciences of CAAS (Chinese Academy of Agricultural Sciences) |
| TR-313 | zxy2006p-1616 | France | Institute of Animal Sciences of CAAS (Chinese Academy of Agricultural Sciences) |
| TR-314 | zxy2006p-1078 | Tajikistan | Institute of Animal Sciences of CAAS (Chinese Academy of Agricultural Sciences) |
| TR-315 | zxy2006p-2552 | Poland | Institute of Animal Sciences of CAAS (Chinese Academy of Agricultural Sciences) |
| TR-316 | zxy2006p-1927 | France | Institute of Animal Sciences of CAAS (Chinese Academy of Agricultural Sciences) |
| TR-317 | zxy2006p-2561 | Poland | Institute of Animal Sciences of CAAS (Chinese Academy of Agricultural Sciences) |
| TR-318 | C 909 | New Zealand | Margot Forde Forage Germplasm Centre (New Zealand) |
| TR-319 | C 922 | Sweden | Margot Forde Forage Germplasm Centre (New Zealand) |
| TR-320 | C 960 | New Zealand | Margot Forde Forage Germplasm Centre (New Zealand) |
| TR-321 | C 1074 | New Zealand | Margot Forde Forage Germplasm Centre (New Zealand) |
| TR-322 | C 1711 | New Zealand | Margot Forde Forage Germplasm Centre (New Zealand) |
| TR-323 | C 1846 | New Zealand | Margot Forde Forage Germplasm Centre (New Zealand) |
| TR-324 | C 2007 | New Zealand | Margot Forde Forage Germplasm Centre (New Zealand) |
| TR-325 | C 2095 | Sweden | Margot Forde Forage Germplasm Centre (New Zealand) |
| TR-326 | C 2096 | Sweden | Margot Forde Forage Germplasm Centre (New Zealand) |
| TR-327 | C 2110 | Sweden | Margot Forde Forage Germplasm Centre (New Zealand) |
| TR-328 | C 2117 | Sweden | Margot Forde Forage Germplasm Centre (New Zealand) |
| TR-329 | C 2387 | England | Margot Forde Forage Germplasm Centre (New Zealand) |
| TR-330 | C 2399 | England | Margot Forde Forage Germplasm Centre (New Zealand) |
| TR-331 | C 2599 | Czech | Margot Forde Forage Germplasm Centre (New Zealand) |
| TR-332 | C 3006 | Chile | Margot Forde Forage Germplasm Centre (New Zealand) |
| TR-333 | C 4953 | Uruguay | Margot Forde Forage Germplasm Centre (New Zealand) |
| TR-334 | C 4959 | New Zealand | Margot Forde Forage Germplasm Centre (New Zealand) |
| TR-335 | C 5000 | New Zealand | Margot Forde Forage Germplasm Centre (New Zealand) |
| TR-336 | C 5879 | Sweden | Margot Forde Forage Germplasm Centre (New Zealand) |
| TR-337 | C 5897 | Italy | Margot Forde Forage Germplasm Centre (New Zealand) |
| TR-338 | C 5997 | Poland | Margot Forde Forage Germplasm Centre (New Zealand) |
| TR-339 | C 6360 | England | Margot Forde Forage Germplasm Centre (New Zealand) |
| TR-340 | C 6362 | England | Margot Forde Forage Germplasm Centre (New Zealand) |
| TR-341 | C 6374 | Scotland | Margot Forde Forage Germplasm Centre (New Zealand) |
| TR-342 | C 6395 | England | Margot Forde Forage Germplasm Centre (New Zealand) |
| TR-343 | C 6404 | England | Margot Forde Forage Germplasm Centre (New Zealand) |
| TR-344 | C 6465 | Sweden | Margot Forde Forage Germplasm Centre (New Zealand) |
| TR-345 | C 6467 | England | Margot Forde Forage Germplasm Centre (New Zealand) |
| TR-346 | C 6468 | England | Margot Forde Forage Germplasm Centre (New Zealand) |
| TR-347 | C 6469 | England | Margot Forde Forage Germplasm Centre (New Zealand) |
| TR-348 | C 6470 | England | Margot Forde Forage Germplasm Centre (New Zealand) |
| TR-349 | C 6472 | England | Margot Forde Forage Germplasm Centre (New Zealand) |
| TR-350 | C 6479 | Uruguay | Margot Forde Forage Germplasm Centre (New Zealand) |
| TR-351 | C 6481 | Belgium | Margot Forde Forage Germplasm Centre (New Zealand) |
| TR-352 | C 6483 | Chile | Margot Forde Forage Germplasm Centre (New Zealand) |
| TR-353 | C 6556 | New Zealand | Margot Forde Forage Germplasm Centre (New Zealand) |
| TR-354 | C 6695 | Romania | Margot Forde Forage Germplasm Centre (New Zealand) |
| TR-355 | C 6696 | Romania | Margot Forde Forage Germplasm Centre (New Zealand) |
| TR-356 | C 6837 | New Zealand | Margot Forde Forage Germplasm Centre (New Zealand) |
| TR-357 | C 6896 | New Zealand | Margot Forde Forage Germplasm Centre (New Zealand) |
| TR-358 | C 7554 | Czech | Margot Forde Forage Germplasm Centre (New Zealand) |
| TR-359 | C 7555 | Czech | Margot Forde Forage Germplasm Centre (New Zealand) |
| TR-360 | C 7556 | Czech | Margot Forde Forage Germplasm Centre (New Zealand) |
| TR-361 | C 7557 | Czech | Margot Forde Forage Germplasm Centre (New Zealand) |
| TR-362 | C 8760 | China | Margot Forde Forage Germplasm Centre (New Zealand) |
| TR-363 | C 8761 | New Zealand | Margot Forde Forage Germplasm Centre (New Zealand) |
| TR-364 | C 9049 | New Zealand | Margot Forde Forage Germplasm Centre (New Zealand) |
| TR-365 | C 9054 | New Zealand | Margot Forde Forage Germplasm Centre (New Zealand) |
| TR-366 | C 9059 | New Zealand | Margot Forde Forage Germplasm Centre (New Zealand) |
| TR-367 | C 9060 | New Zealand | Margot Forde Forage Germplasm Centre (New Zealand) |
| TR-368 | C 9061 | New Zealand | Margot Forde Forage Germplasm Centre (New Zealand) |
| TR-369 | C 9265 | New Zealand | Margot Forde Forage Germplasm Centre (New Zealand) |
| TR-370 | C 9345 | China | Margot Forde Forage Germplasm Centre (New Zealand) |
| TR-371 | C 10207 | New Zealand | Margot Forde Forage Germplasm Centre (New Zealand) |
| TR-372 | C 10611 | Brazil | Margot Forde Forage Germplasm Centre (New Zealand) |
| TR-373 | C 10612 | Brazil | Margot Forde Forage Germplasm Centre (New Zealand) |
| TR-374 | C 11578 | New Zealand | Margot Forde Forage Germplasm Centre (New Zealand) |
| TR-375 | C 11698 | China | Margot Forde Forage Germplasm Centre (New Zealand) |
| TR-376 | C 13013 | New Zealand | Margot Forde Forage Germplasm Centre (New Zealand) |
| TR-377 | C 13331 | Sweden | Margot Forde Forage Germplasm Centre (New Zealand) |
| TR-378 | C 14276 | New Zealand | Margot Forde Forage Germplasm Centre (New Zealand) |
| TR-379 | C 14278 | China | Margot Forde Forage Germplasm Centre (New Zealand) |
| TR-380 | C 14282 | New Zealand | Margot Forde Forage Germplasm Centre (New Zealand) |
| TR-381 | C 14317 | Greece | Margot Forde Forage Germplasm Centre (New Zealand) |
| TR-382 | C 14318 | Greece | Margot Forde Forage Germplasm Centre (New Zealand) |
| TR-383 | C 15117 | Spain | Margot Forde Forage Germplasm Centre (New Zealand) |
| TR-384 | C 15120 | New Zealand | Margot Forde Forage Germplasm Centre (New Zealand) |
| TR-385 | C 15872 | Czech | Margot Forde Forage Germplasm Centre (New Zealand) |
| TR-386 | C 15873 | Czech | Margot Forde Forage Germplasm Centre (New Zealand) |
| TR-387 | C 15968 | Japan | Margot Forde Forage Germplasm Centre (New Zealand) |
| TR-388 | C 15973 | Slovakia | Margot Forde Forage Germplasm Centre (New Zealand) |
| TR-389 | C 16027 | New Zealand | Margot Forde Forage Germplasm Centre (New Zealand) |
| TR-390 | C 16042 | Ireland | Margot Forde Forage Germplasm Centre (New Zealand) |
| TR-391 | C 16561 | Canada | Margot Forde Forage Germplasm Centre (New Zealand) |
| TR-392 | C 16562 | Canada | Margot Forde Forage Germplasm Centre (New Zealand) |
| TR-393 | C 16563 | Canada | Margot Forde Forage Germplasm Centre (New Zealand) |
| TR-394 | C 17950 | New Zealand | Margot Forde Forage Germplasm Centre (New Zealand) |
| TR-395 | C 18725 | New Zealand | Margot Forde Forage Germplasm Centre (New Zealand) |
| TR-396 | C 18954 | New Zealand | Margot Forde Forage Germplasm Centre (New Zealand) |
| TR-397 | C 18966 | New Zealand | Margot Forde Forage Germplasm Centre (New Zealand) |
| TR-398 | C 18969 | New Zealand | Margot Forde Forage Germplasm Centre (New Zealand) |
| TR-399 | C 19736 | New Zealand | Margot Forde Forage Germplasm Centre (New Zealand) |
| TR-400 | C 20130 | New Zealand | Margot Forde Forage Germplasm Centre (New Zealand) |
| TR-401 | C 20131 | New Zealand | Margot Forde Forage Germplasm Centre (New Zealand) |
| TR-402 | C 21223 | China | Margot Forde Forage Germplasm Centre (New Zealand) |
| TR-403 | C 21516 | New Zealand | Margot Forde Forage Germplasm Centre (New Zealand) |
| TR-404 | C 21517 | New Zealand | Margot Forde Forage Germplasm Centre (New Zealand) |
| TR-405 | C 21519 | New Zealand | Margot Forde Forage Germplasm Centre (New Zealand) |
| TR-406 | C 21520 | New Zealand | Margot Forde Forage Germplasm Centre (New Zealand) |
| TR-407 | C 21908 | New Zealand | Margot Forde Forage Germplasm Centre (New Zealand) |
| TR-408 | C 22941 | New Zealand | Margot Forde Forage Germplasm Centre (New Zealand) |
| TR-409 | C 22942 | New Zealand | Margot Forde Forage Germplasm Centre (New Zealand) |
| TR-410 | C 23249 | New Zealand | Margot Forde Forage Germplasm Centre (New Zealand) |
| TR-411 | C 23434 | New Zealand | Margot Forde Forage Germplasm Centre (New Zealand) |
| TR-412 | C 23842 | New Zealand | Margot Forde Forage Germplasm Centre (New Zealand) |
| TR-413 | C 23851 | New Zealand | Margot Forde Forage Germplasm Centre (New Zealand) |
| TR-414 | C 24195 | New Zealand | Margot Forde Forage Germplasm Centre (New Zealand) |
| TR-415 | C 24196 | New Zealand | Margot Forde Forage Germplasm Centre (New Zealand) |
| TR-416 | C 24294 | New Zealand | Margot Forde Forage Germplasm Centre (New Zealand) |
| TR-417 | C 24296 | New Zealand | Margot Forde Forage Germplasm Centre (New Zealand) |
| TR-418 | C 24693 | New Zealand | Margot Forde Forage Germplasm Centre (New Zealand) |
| TR-419 | C 24694 | New Zealand | Margot Forde Forage Germplasm Centre (New Zealand) |
| TR-420 | C 24695 | New Zealand | Margot Forde Forage Germplasm Centre (New Zealand) |
| TR-421 | C 24970 | New Zealand | Margot Forde Forage Germplasm Centre (New Zealand) |
| TR-422 | C 24972 | New Zealand | Margot Forde Forage Germplasm Centre (New Zealand) |
| TR-423 | C 25073 | New Zealand | Margot Forde Forage Germplasm Centre (New Zealand) |
| TR-424 | C 25074 | New Zealand | Margot Forde Forage Germplasm Centre (New Zealand) |
| TR-425 | C 25179 | New Zealand | Margot Forde Forage Germplasm Centre (New Zealand) |
| TR-426 | C 25286 | New Zealand | Margot Forde Forage Germplasm Centre (New Zealand) |
| TR-427 | C 25302 | New Zealand | Margot Forde Forage Germplasm Centre (New Zealand) |
| TR-428 | C 25421 | Lithuania | Margot Forde Forage Germplasm Centre (New Zealand) |
| TR-429 | C 25424 | Finland | Margot Forde Forage Germplasm Centre (New Zealand) |
| TR-430 | C 25427 | Switzerland | Margot Forde Forage Germplasm Centre (New Zealand) |
| TR-431 | C 25428 | Switzerland | Margot Forde Forage Germplasm Centre (New Zealand) |
| TR-432 | C 25491 | Poland | Margot Forde Forage Germplasm Centre (New Zealand) |
| TR-433 | C 25495 | Estonia | Margot Forde Forage Germplasm Centre (New Zealand) |
| TR-434 | C 25513 | New Zealand | Margot Forde Forage Germplasm Centre (New Zealand) |
| TR-435 | C 25515 | Norway | Margot Forde Forage Germplasm Centre (New Zealand) |
| TR-436 | C 26456 | Uruguay | Margot Forde Forage Germplasm Centre (New Zealand) |
| TR-437 | C 26501 | New Zealand | Margot Forde Forage Germplasm Centre (New Zealand) |
| TR-438 | C 26502 | New Zealand | Margot Forde Forage Germplasm Centre (New Zealand) |
| TR-439 | C 26792 | New Zealand | Margot Forde Forage Germplasm Centre (New Zealand) |
| TR-440 | C 26794 | New Zealand | Margot Forde Forage Germplasm Centre (New Zealand) |
| TR-441 | C 26795 | New Zealand | Margot Forde Forage Germplasm Centre (New Zealand) |
| TR-442 | C 27047 | New Zealand | Margot Forde Forage Germplasm Centre (New Zealand) |
| TR-443 | C 27069 | New Zealand | Margot Forde Forage Germplasm Centre (New Zealand) |
| TR-444 | C 27071 | New Zealand | Margot Forde Forage Germplasm Centre (New Zealand) |
| TR-445 | C 27073 | New Zealand | Margot Forde Forage Germplasm Centre (New Zealand) |
| TR-446 | C 27908 | New Zealand | Margot Forde Forage Germplasm Centre (New Zealand) |
| TR-447 | C 28153 | New Zealand | Margot Forde Forage Germplasm Centre (New Zealand) |
| TR-448 | C 24404 | New Zealand | Margot Forde Forage Germplasm Centre (New Zealand) |
