# Supplementary material for: Genetic diversity and population structure analysis in a large collection of white clover (Trifolium repens L.) germplasm worldwide
Source: PeerJ. 2021 May 3;9:e11325. doi: 10.7717/peerj.11325 (PMC8101478; doi:10.7717/peerj.11325)
Supplement: Table S2 [file peerj-09-11325-s002.docx]

| SSR name | LG | cM | Type | Motif | Repeats | eSize (bp) | Forward primer (5'-3') | Reverse primer (5'-3') |
| --- | --- | --- | --- | --- | --- | --- | --- | --- |
| ats002 | Tr_3-1 | 87.357 | tri | atg | 8 | 201 | GGCGAGAGTGAGACAGTGGA | TGTCGCTCCAACCACCAGTA |
| gtrs1113 | Tr_5-1 | 44.93 | tri | aat | 8 | 169 | ATTTGGCCTCTTGTGTCAAGTAA | TGATGTTGGTGACTTATTATTGTGC |
| gtrs165 | Tr_8-1 | 51.928 | tri | aca | 8 | 122 | GATGAAGCTGAAAGATGGATTTG | ATTGATTTGGGTCTACGTTGTTG |
| gtrs171 | Tr_8-1 | 19.672 | tri | atc | 7 | 103 | GATAGCTTCATCAACATGGAACA | CTTCTTCATTCACCACAAAGTGA |
| gtrs173 | Tr_8-1 | 39.019 | tri | atc | 9 | 123 | GATCTCTCCTTCCCACTCAACTA | TGGTGATGACAATGAGGAAGAAT |
| gtrs211 | Tr_3-2 | 81.42 | tri | tct | 9 | 128 | TGAGACCTCCCAATAACATTCAC | CTCCGAAGCTCACTTGGTTAGTA |
| gtrs242 | Tr_1-1 | 31.12 | tri | aac | 7 | 174 | CACCAGAACAGAACTCAATCCTC | TCGTTTGATTCGATTTGAGAGTT |
| gtrs285 | Tr_6-2 | 3.077 | tri | ctg | 9 | 171 | GAAACCCATTTCACCAAGAAAC | ATAGAGCACCAAACCAGTTGAAA |
| gtrs292 | Tr_8-1 | 48.064 | tri | gcg | 7 | 186 | TCCAAACAGATCAAATTATTCACG | CATCGCAACTCAACCTTCTAAA |
| gtrs319 | Tr_7-1 | 20.855 | tetra | acga | 9 | 199 | CTCACGTTCATTCACACAACAAC | CTGATTAGACCTTTGTCGCATTT |
| gtrs371 | Tr_8-1 | 43.091 | tri | cat | 7 | 264 | TATCACCACCGACACAGTAATTC | TTTCAAATTCCTAACATGGCAAA |
| gtrs376 | Tr_1-2 | 63.048 | tri | ctt | 10 | 311 | TGAAGAATCAATCTGAAATCCAA | AGGGTTTATGTTGAGGGATAGGA |
| gtrs541 | Tr_8-2 | 5.561 | tri | aac | 7 | 113 | CATTCTCTTCCATAAACATTTCCA | AAGAGGTTGAGGAGTTTGAGGTT |
| gtrs564 | Tr_1-2 | 84.147 | tri | caa | 7 | 120 | AACAATGGTGTCTTACCCAGAAA | AGTAGTTGTTGTTGTTGAAGATGAA |
| gtrs591 | Tr_5-2 | 35.358 | tri | tca | 7 | 107 | TCAAGATCAAGAACCCACATTCT | TGTTGATGCTGTAATGGTTTGAG |
| gtrs679 | Tr_4-2 | 59.02 | tri | taa | 7 | 225 | GGACTCTCACTGCTTTCTGCTAC | TGAGGAAGAATTTGAACTTGGAA |
| gtrs701 | Tr_6-1 | 2.707 | tri | ttg | 8 | 177 | AGCTTACAGTGCAAAGAAGGTTG | ACCAACAAAGTCCCACTCACAT |
| gtrs723 | Tr_5-2 | 21.9 | tri | cca | 7 | 261 | TGGAGCTGGATACAAAGAAGAAG | GACGAGTCCGAGGAAGAGTATG |
| gtrs749 | Tr_7-1 | 7.509 | tri | att | 8 | 265 | CCAAATCATACACACATCACCAC | TTCCTCACACCCTCTCTTATTGA |
| gtrs760 | Tr_3-2 | 77.151 | tri | ctt | 7 | 257 | AGGACAAGTTAAAGCCACCTTTC | TTGTTGATGCTACTCCTGAGGTT |
| gtrs851 | Tr_1-2 | 8.748 | tri | caa | 8 | 328 | AGTCTCCTTGAAGATACGGAACC | GCGAAATTGAAAGATTTAGCAGA |
| gtrs949 | Tr_4-1 | 80.816 | tetra | cata | 6 | 206 | TATGGATGGAATGGTAATTTGGA | CGCGTAAGCAATAATAGTGTGAG |
