# Supplementary figures and images for: Genetic diversity and population structure analysis in a large collection of white clover (Trifolium repens L.) germplasm worldwide
Source: PeerJ. 2021 May 3;9:e11325. doi: 10.7717/peerj.11325 (PMC8101478; doi:10.7717/peerj.11325)

**A**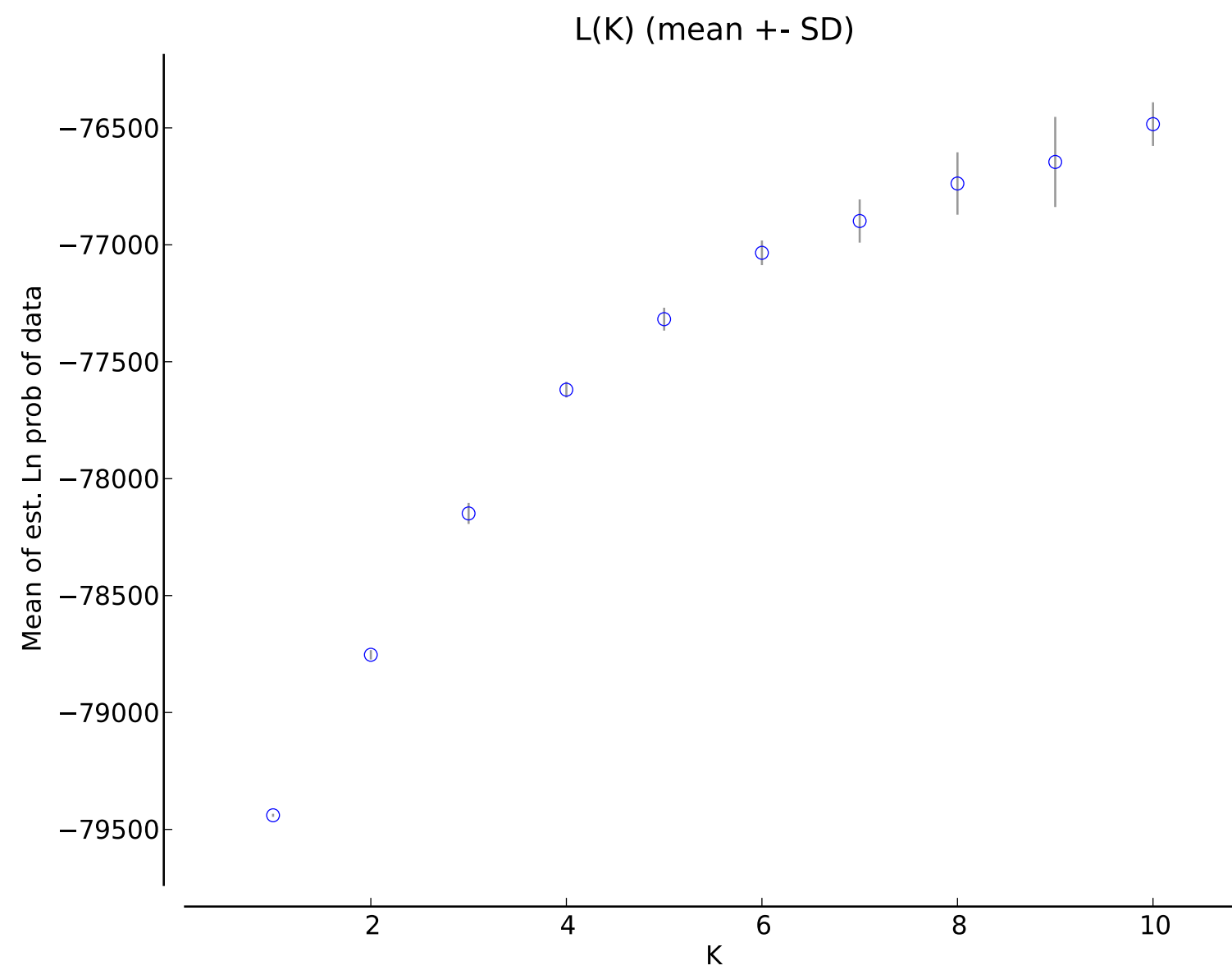**B**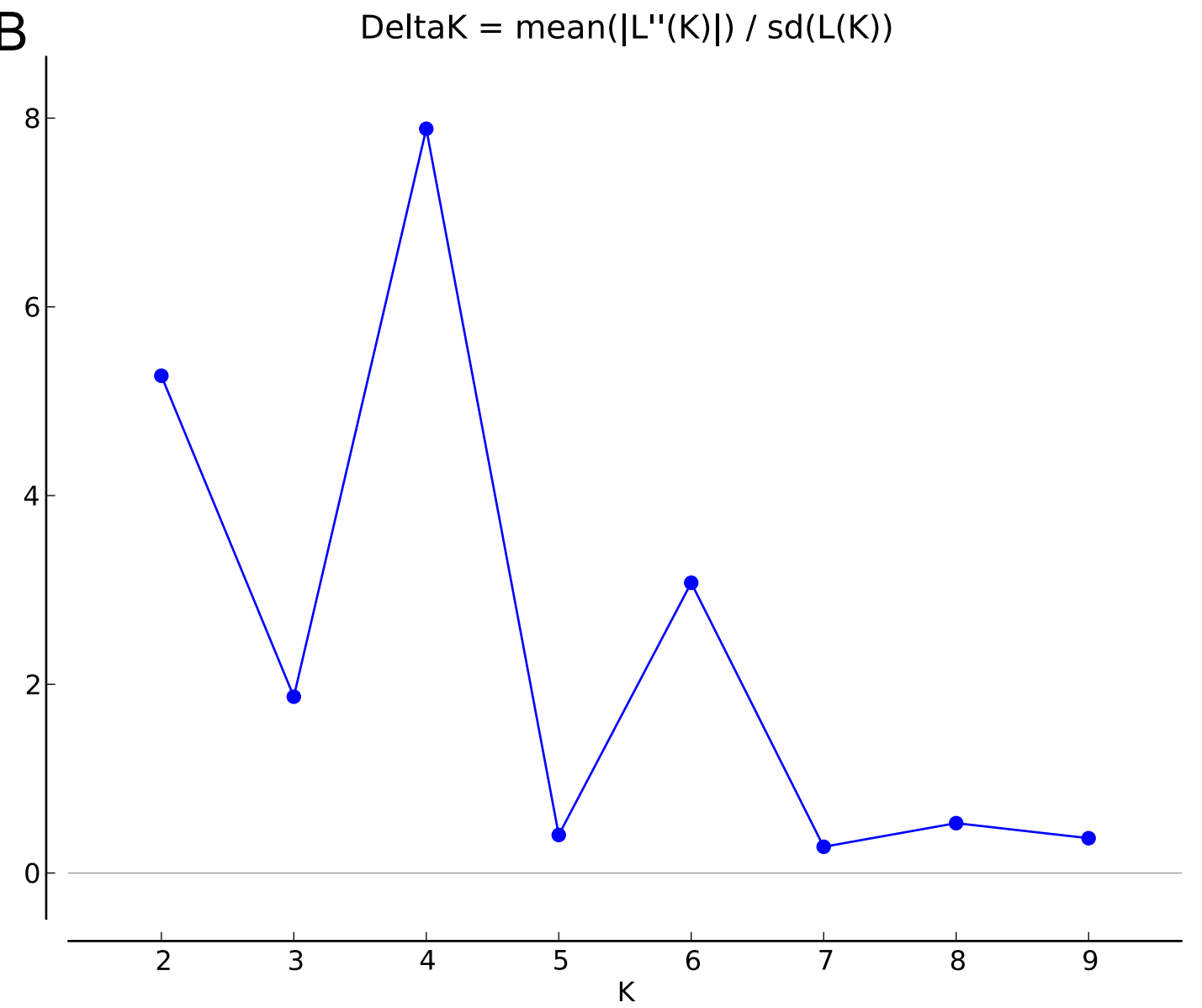

Supplement: Figure S1 [file peerj-09-11325-s004.pdf]

A

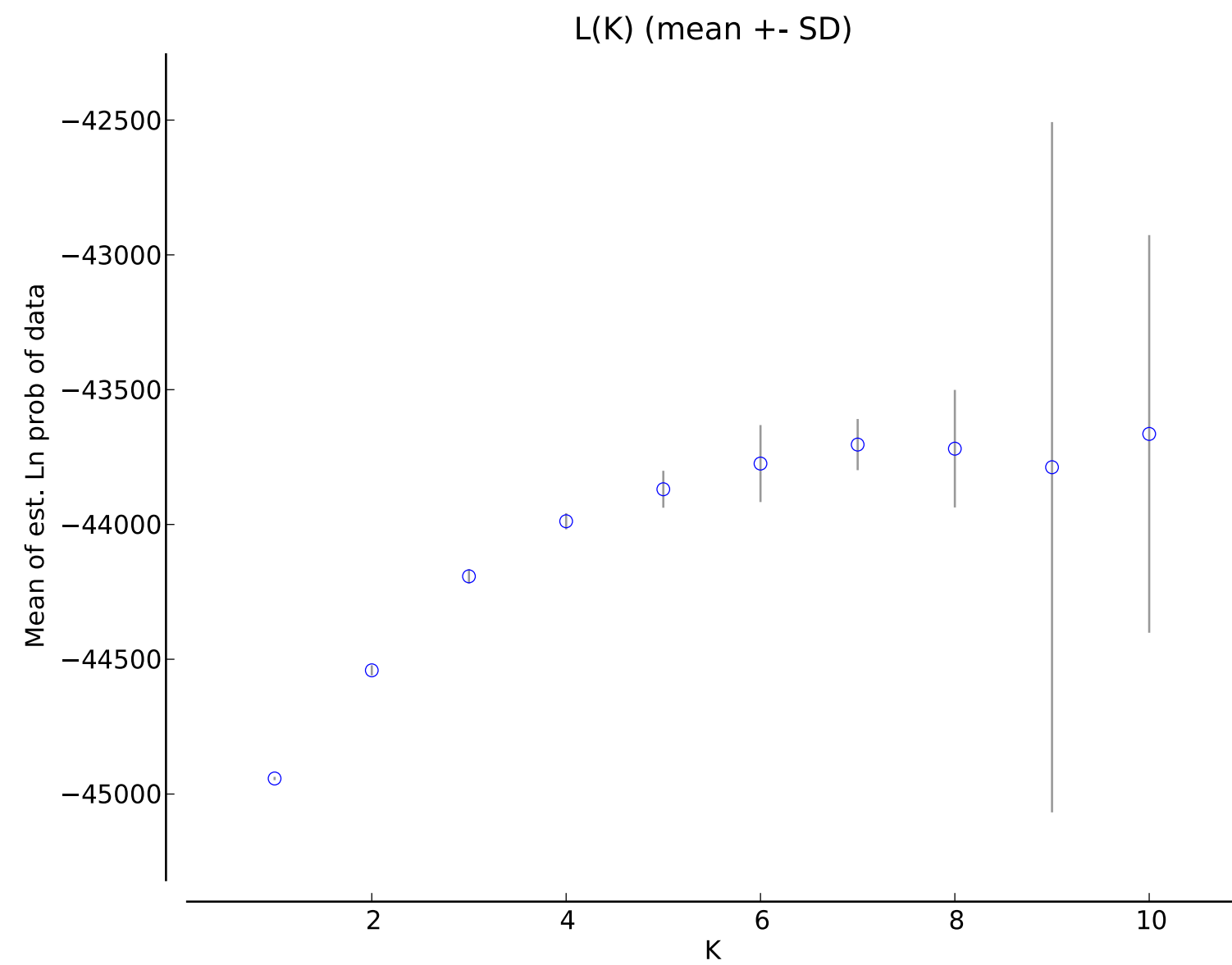

B

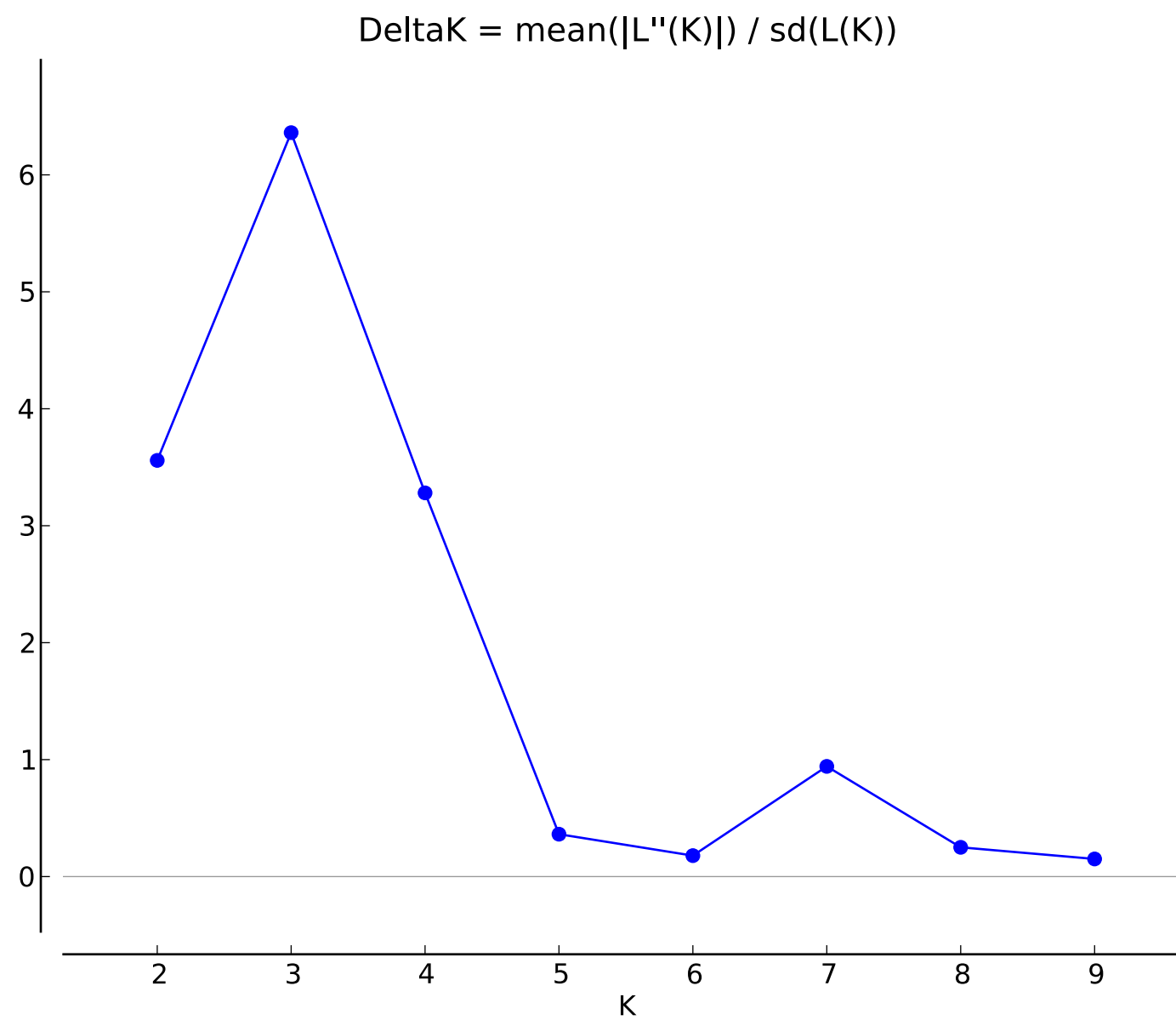

Supplement: Figure S2 [file peerj-09-11325-s005.pdf]

**A**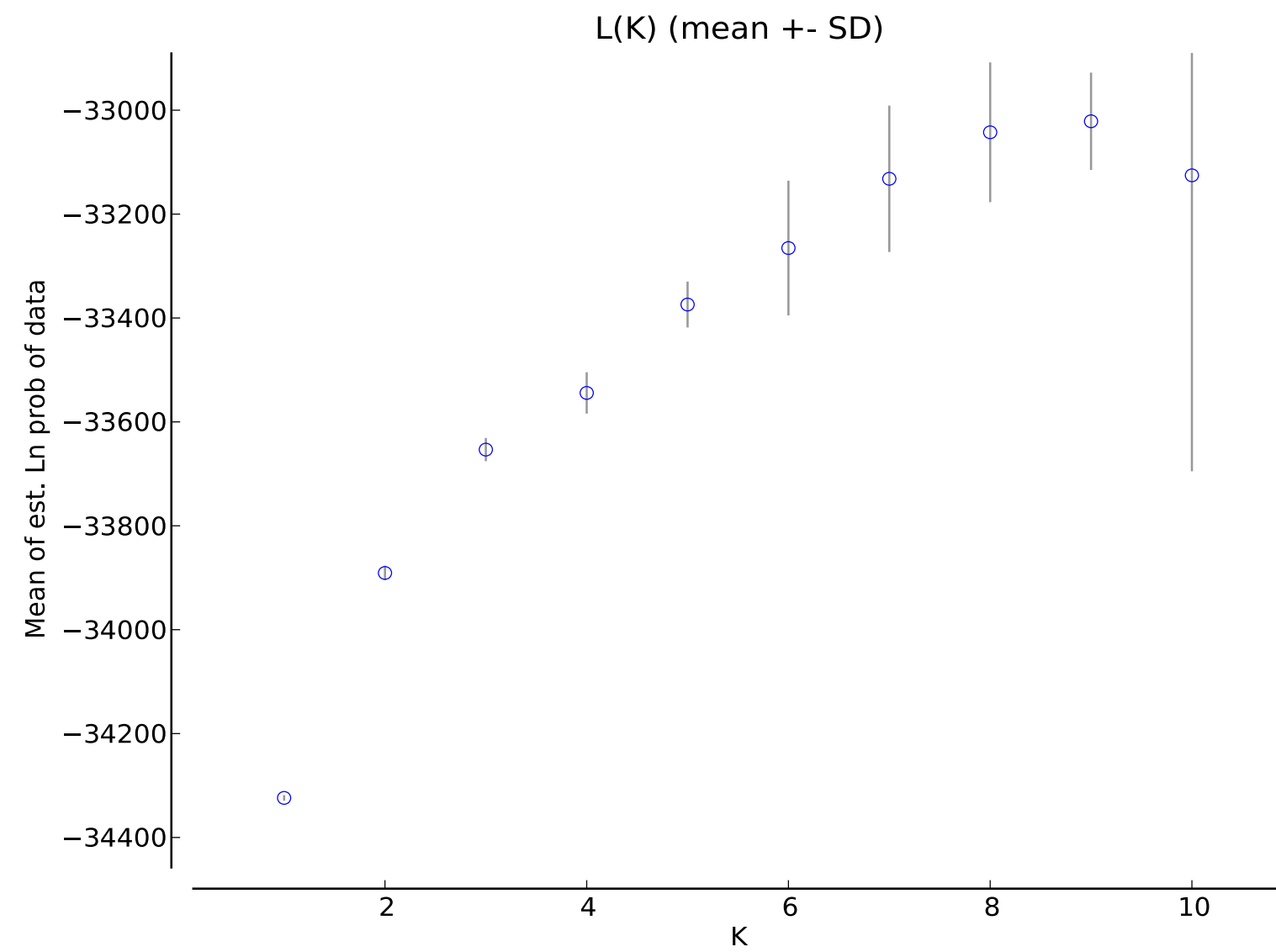**B**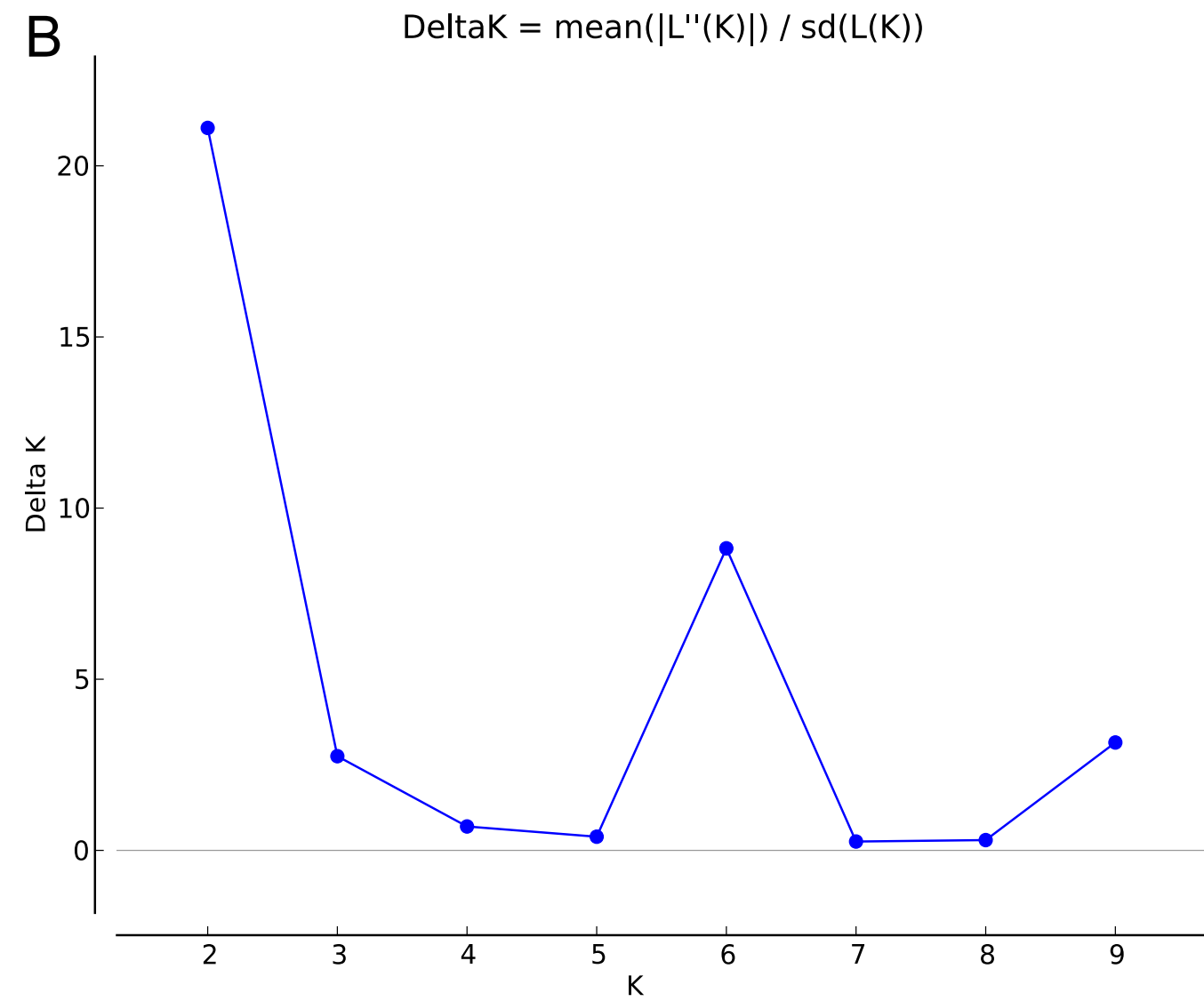

Supplement: Figure S3 [file peerj-09-11325-s006.pdf]
